# S-MGBs bearing amidine tail groups are potent, selective antiplasmodial agents†

**DOI:** 10.1039/d4md00619d

**Published:** 2024-10-16

**Authors:** Marina Perieteanu, Tayner Rodriguez Garzon, Leah M. C. McGee, Abedawn I. Khalaf, Colin J. Suckling, Rebecca Beveridge, Vicky M. Avery, Fraser J. Scott

**Affiliations:** a Department of Pure and Applied Chemistry, University of Strathclyde Glasgow UK fraser.j.scott@strath.ac.uk; b Discovery Biology, Centre for Cellular Phenomics, Griffith University Nathan Queensland 4111 Australia; c School of Environment & Sciences, Griffith University Nathan Queensland 4111 Australia

## Abstract

There were an estimated 249 million cases of malaria globally in 2022, causing approximately 608 000 deaths. Most of these are attributed to infection by *P. falciparum*. Strathclyde minor groove binders (S-MGBs) are a promising new class of anti-infective agent that have been shown to be effective against many infectious organisms, including *P. falciparum*. A panel of 25 S-MGBs was synthesised, including those bearing an amidine tail group, and their antiplasmodial activity against 3D7 and Dd2 strains was determined *in vitro* using an asexual *P. falciparum* imaging assay. Determination of activity against HEK293 cells allowed for selective cytotoxicity to be measured. DNA binding studies were carried out using native mass spectrometry and DNA thermal shift assays. A comparison of 3D7 (chloroquine sensitive) and Dd2 (chloroquine resistant) potency showed no evidence of cross-resistance across the S-MGB set. **S-MGB-356**, **S-MGB-368** and **S-MGB-359**, amidine tail containing S-MGBs, were identified as the most promising hit compounds based on their selectivity indices (HEK293/3D7) of >612.6, >335.8 and >264.8, respectively. **S-MGB-356**, **S-MGB-368** and **S-MGB-359** were confirmed to bind to DNA as dimers, with gDNA thermal shifts (Δ*T*_m_) of 12 °C, 3 °C and 16 °C, respectively. Together, these data demonstrate that amidine tail bearing S-MGBs are promising hit compounds against *P. falciparum*, and can be further optimised into lead compounds.

## Abstract 2

25 S-MGBs, including those bearing an amidine tail group, were investigated for their antiplasmodial, *in vitro* activity against 3D7 and Dd2 strains of *P. falciparum*. Several potent, and selective, hit compounds were identified.

## Graphical abstract

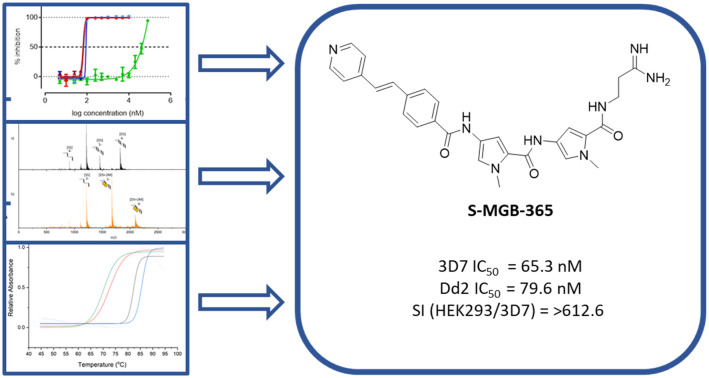

## 1. Introduction

Malaria has major health, economic and societal impacts, with an estimated 249 million cases globally in 2022 resulting in ∼608 000 deaths, the majority of which are attributed to *P. falciparum*.^1^ Whilst a steady decline in the number of deaths was observed between 2000 and 2019, current data suggests this is no longer the case,^1^ with the emergence of drug resistance and the impact of COVID-19 being major contributing factors.^2^

Parasite resistance has emerged to all current antiplasmodial drug classes, including the front-line artemisinin combination therapies.^3^ Recently, several new small molecules have progressed through to clinical trials or *in vivo* studies (reviewed in [2]), however concerns remain due to the rapid acquisition of resistance reported in the last decade. The scope of drug discovery efforts needs to broaden to encompass molecules with alternative mechanisms of action, particularly those less susceptible to drug resistance, to address the current situation.

Minor groove binders (MGBs) are a class of small molecules that exert their biological effects through binding to the minor groove of double stranded DNA (dsDNA). This binding event disrupts the normal processing of the DNA through either direct occlusion, or indirect topological changes, of a DNA-protein interaction.^4^ Several structural subclasses of MGB exist, such as the diamidines, which includes the antiparasitic compounds diminazene, a treatment for animal African trypanosomiasis, and pentamidine, used in the treatment of human African trypanosomiasis. Another subclass of MGB are the polyamides, which are derived from the natural product distamycin. Many distamycin analogues have been shown to have potent *in vitro* and *in vivo* antiparasitic activities, including those discovered at the University of Strathclyde, termed Strathclyde MGBs (S-MGBs).^5^ Indeed, members of both the polyamides and diamidines have been shown to have some *in vitro* antiplasmodial activity.^6^

As is typical for distamycin analogues, S-MGBs have been shown to interact strongly with many AT-rich sequences of dsDNA, resulting in a multi-targeted anti-infective drug (MTAID) approach in their design.^7^ This multi-targeted approach has been demonstrated to reduce the likelihood of the generation of target-based resistance in pathogens, and has also enabled a broad-spectrum of activity across different phylogenetic groups of pathogens *viz.* bacteria, fungi, viruses and parasites.^5^

In the antiparasitic context, S-MGBs have been shown to be effective at curing infection in *in vivo* models of animal African trypanosomiasis, caused by *Trypanosoma congolense*.^8^ We have also previously reported the *in vitro* antiplasmodial activity of a set of S-MGBs, in which we showed that compounds bearing an alkene link to the head group, as opposed to an amide or amidine, were generally more potent.^9^ In particular, we identified compound **S-MGB-169** (named compound 27 in ref. 9), bearing an alkene-linked head group and a morpholine tail group, with a selectivity index of >514 as being of interest. Since then, the S-MGB drug discovery platform has significantly expanded to include compounds with more diverse structures. Many S-MGBs bear an amidine tail group, rather than the weakly basic morpholine tail group, as we have shown that this change gives rise to lower cytotoxicity through lower intracellular accumulation in mammalian cells, and enhanced solubility.^10^

In this study, we have screened a diverse set of 25 S-MGBs, crucially including strong representation of the more contemporary amidine tail group, against *P. falciparum*. We have again shown that S-MGBs are not affected by existing resistance mechanisms, by comparing activity against *P. falciparum* 3D7 and Dd2 strains. We have also shown that the amidine tail group is an important driver of selectivity in antiplasmodial S-MGBs. The compounds, **S-MGB-365**, **S-MGB-368** and **S-MGB-359** were demonstrated to bind to dsDNA, and emerge as top candidates for future drug development activities.

## 2. Results and discussion

### 2.1 S-MGB library sampling and compound synthesis

**Fig. 1:**
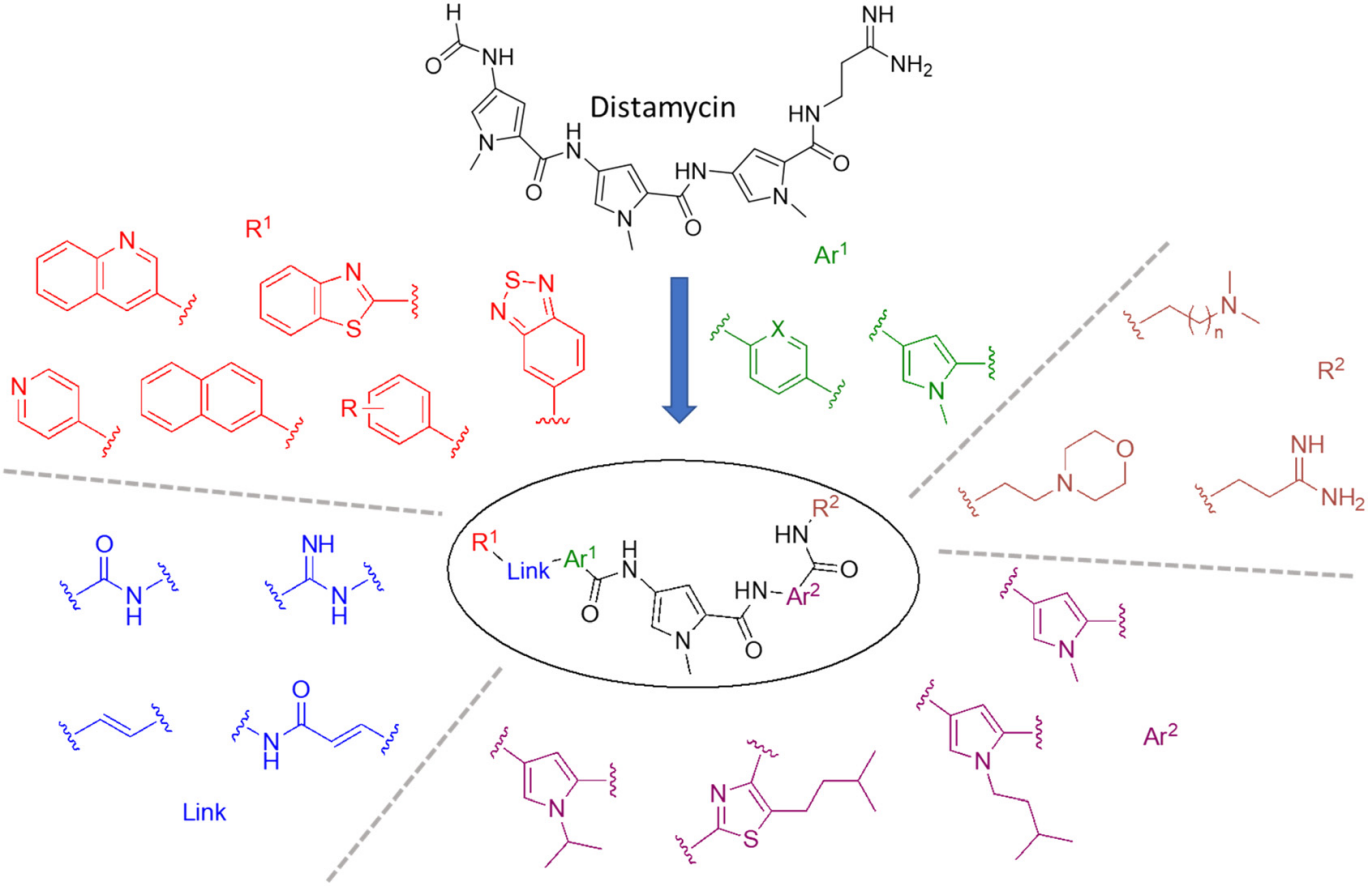
Distamycin and the variety of S-MGBs examined in this study. For *R*^1^, *R* = *m*-OMe, *p*-CF_3_, *p*-N(Me)_2_, *o*-OH, *p*-OMe, *p*-F, *m*-F, *m*-CF_3_. For *R*^2^, *n* = 1 or 2. Ar^1^ X = C or N.

**Scheme 1:**
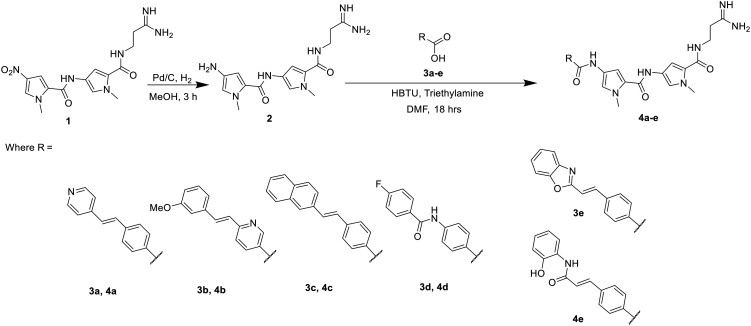
Synthesis of 5 novel S-MGBs, **S-MGB-365**, **S-MGB-368**, **S-MGB-359**, **S-MGB-388** and **S-MGB-361** (4a–e, respectively).

A representative sample of compounds selected from our S-MGB library, covered most alterations of the head, tail, side chains and body of the distamycin template, including the more contemporary amidine tail group. This generated a diverse set of 25 compounds (summary structures in Fig. 1; full list of structures in Table S1†). Of this set, the synthesis of five S-MGBs has not previously been reported: **S-MGB-365**, **S-MGB-368**, **S-MGB-359**, **S-MGB-388** and **S-MGB-361**, 4a–e, respectively (Scheme 1). The synthesis of these new compounds followed our typical S-MGB synthetic strategy. Briefly, the nitro moiety of the tail group dimer, 1, was hydrogenated with Pd/C affording the corresponding amine, 2, which was used without isolation in a HBTU-mediated coupling with the head group dimer carboxylic acids, 3a–e, to give final S-MGBs, 4a–e (Scheme 1). It should be noted, that as previously reported,^11^ upon coupling conditions, 3e, ring opens and thus during purification the corresponding 4e, **S-MGB-361**, is obtained.

### 2.2 *In vitro* potency evaluation

All compounds were evaluated in *in vitro* asexual *P. falciparum* assays against 3D7, and Dd2, allowing for an assessment of cross-resistance (Table 1). The data is highly reproducible across both parasite strains. Additionally, cytotoxicity was measured against HEK293 cells, and the selectivity index was derived from the ratio of HEK293 IC_50_ to *P. falciparum* 3D7 IC_50_. Where an accurate IC_50_ value was not obtained, the selectivity indices were estimated using the relative IC_50_ value and defined as ≥ to provide a conservative comparison of the activity levels obtained. All compounds evaluated were greater than 95% pure by HPLC and ^1^H NMR.

**Table 1:** S-MGB IC_50_ (nM) against *P. falciparum* 3D7 and Dd2, and HEK293, ordered by selectivity index. Concentration response curves generated in 11-point dose response, *N* = 2 duplicate point against both *P. falciparum* 3D7 and Dd2

S-MGB code	3D7 IC_50_ (nM)	Dd2 IC_50_ (nM)	Dd2/3D7	HEK293 (IC_50_ (nM) or % inhibition at 80 μM)	aHEK 293 C_50_ value M	SI, HEK293/3D7
**365** c	65.3 ± 0.2	79.6 ± 0.2	1.2	95%	40 000	>612.6
**368** c	134.0 ± 0.3	201.7 ± 0.3	1.5	92%	45 000	>335.8
**359** c	302.1 ± 0.6	318.6 ± 1.1	1.1	23%	80 000^b^	>264.8
**337**	95.6 ± 0.0	101.8 ± 0.2	1.1	101%	21 000	>219.7
**131**	200.1 ± 0.0	254.4 ± 0.2	1.3	101%	35 000	>194.9
**188**	57.0 ± 0.1	61.9 ± 0.3	1.1	86%	11 000	>193
**388** c	181.4 ± 2.2	228.6 ± 2.0	1.3	93%	35 000	>192.9
**247**	108.9 ± 0.2	138.6 ± 0.2	1.3	100%	20 000	>183.7
**386**	254.9 ± 1.2	207.1 ± 1.3	0.8	101%	40 000	>157
**380**	74.7 ± 2.8	83.9 ± 3.6	1.1	8203	—	110
**367** c	367.3 ± 0.9	620.3 ± 0.3	1.4	81%	40 000	>108.9
**330**	407.7 ± 0.1	636.1 ± 0.4	1.6	100%	40 000	>98.1
**248**	33.4 ± 0.7	57.0 ± 0.6	1.7	3134	—	94
**246**	43.0 ± 0.6	53.0 ± 0.4	1.2	3856	—	90
**390** c	532.6 ± 0.9	640.2 ± 0.6	1.2	78%	40 000	>75.1
**378**	150.1 ± 4.0	170.6 ± 2.9	1.1	10 453	—	70
**361** c	633.9 ± 0.2	558.5 ± 0.4	0.9	90%	40 000	>63.1
**376**	147.2 ± 1.4	135.1 ± 1.6	0.9	9100	—	62
**391** c	671.3 ± 1.4	1295.5 ± 0.7	1.9	100%	40 000	>59.6
**377**	73.1 ± 4.7	105.2 ± 6.3	1.4	4301	—	59
**379**	149.5 ± 2.7	148.3 ± 5.4	1.0	8337	—	56
**176**	756.0 ± 0.8	1236.0 ± 3.0	1.6	80%	40 000	>53
**389** c	857.5 ± 1.3	488.0 ± 1.8	0.6	92%	40 000	>46.6
**192**	96.3 ± 12.8	108.0 ± 20.6	1.1	3188	—	33
**245**	161.0 ± 0.5	169.1 ± 0.5	1.1	2747	—	17
**Chloroquine**	31.3 ± 9.7	224.6 ± 84.4	7.2	14%		>2500
**Puromycin**	41.9 ± 7.2	49.3 ± 3.5	1.2	377.8		9
**Pyrimeth-amine**	2.69 ± 0.26	>80 000	>29 740	3977		1478
**Pyronaridine**	26.7 ± 3.8	33.3 ± 6.1	1.3	3050		114

a Relative HEK 293 IC_50_ value used to calculate SI where accurate IC_50_ not available.

b Top concentration tested, not rIC50 as < 30% inhibition thus SI calculated with the top concentration.

c Compounds bearing an amidine tail group.

The compounds in this set possess a range of potency and cytotoxicity, enabling an assessment of structure–activity relationships. Given the potential for DNA targeting compounds to be indiscriminately cytotoxic, we first assessed selectivity between 3D7 potency and HEK293 cytotoxicity. Almost all the S-MGBs showed excellent selectivity for *P. falciparum*, compared with HEK293 cells, with selectivity indices ranging between 17 and >612.6 (Table 1). This suggests that the biological mechanisms governing potency and/or uptake in *P. falciparum* are sufficiently different to those in HEK293 to allow for selectivity.

**Fig. 2:**
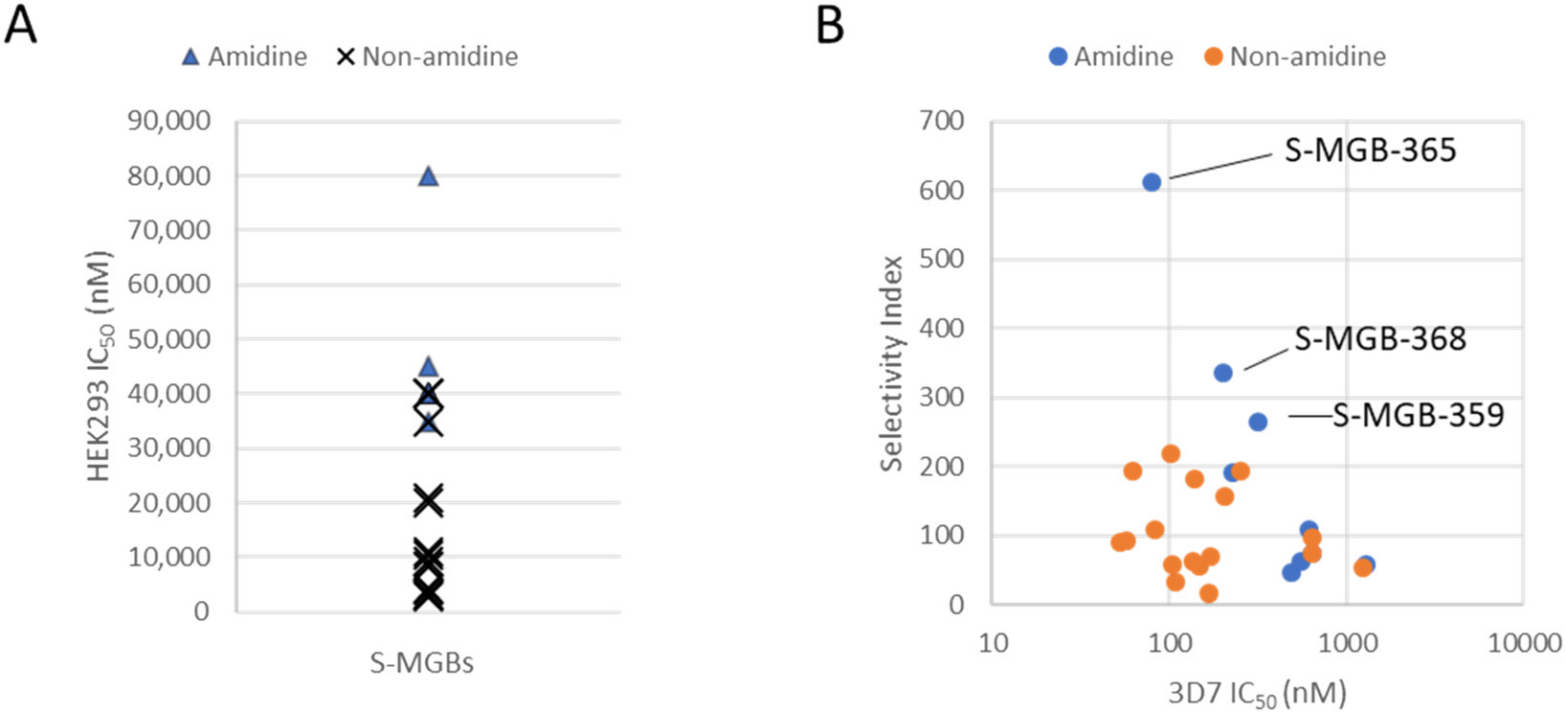
Panel A, a comparison of the cytotoxicity amidine tail and non-amidine tail S-MGBs. Panel B, selectivity index (HEK293/3D7) compared to potency (3D7) of S-MGBs to identify three most promising compounds, **S-MGB-365** (4a), **S-MGB-368** (4b) and **S-MGB-359** (4c).

Our previous observation of amidine tail S-MGBs having lower cytotoxicity holds true in this data set.^10^ When the cytotoxicity data are grouped into either amidine or non-amidine tail group containing S-MGBs, the amidine tail group compounds are found at the low cytotoxicity end of the rank ordering (Fig. 2, Panel A). Indeed, no amidine tail group compounds appear in the high cytotoxicity half of the rank ordered data. Furthermore, when taking into account potency, 3 compounds from the amidine tail group set (4a, **S-MGB-365**; 4b, **S-MGB-368**; and 4c, **S-MGB-359**) have the most promising Compared to the reference compounds, chloroquineCompared to the reference compounds, chloroquineselectivity indices (Fig. 2, Panel B).

Compared to the reference compounds, chloroquine, puromycin, pyrimethamine and pyronaridine, S-MGBs are a promising series. We considered the Dd2/3D7 potency, and with values not deviating far from 1, there is no evidence of cross-resistance across the S-MGBs. This contrasts to chloroquine, with a ratio of 7.2 and pyrimethamine with a ratio >372. Neither puromycin nor pyronaridine are affected by cross-resistance, however, their selectivity indices are 9 and 114, respectively, lower than several of the S-MGBs in this data set. Indeed, the potency of S-MGB-365 (65.3 nM) is on the same order of magnitude as puromycin (41.9 nM) and pyronaridine (26.7 nM).

**Fig. 3:**
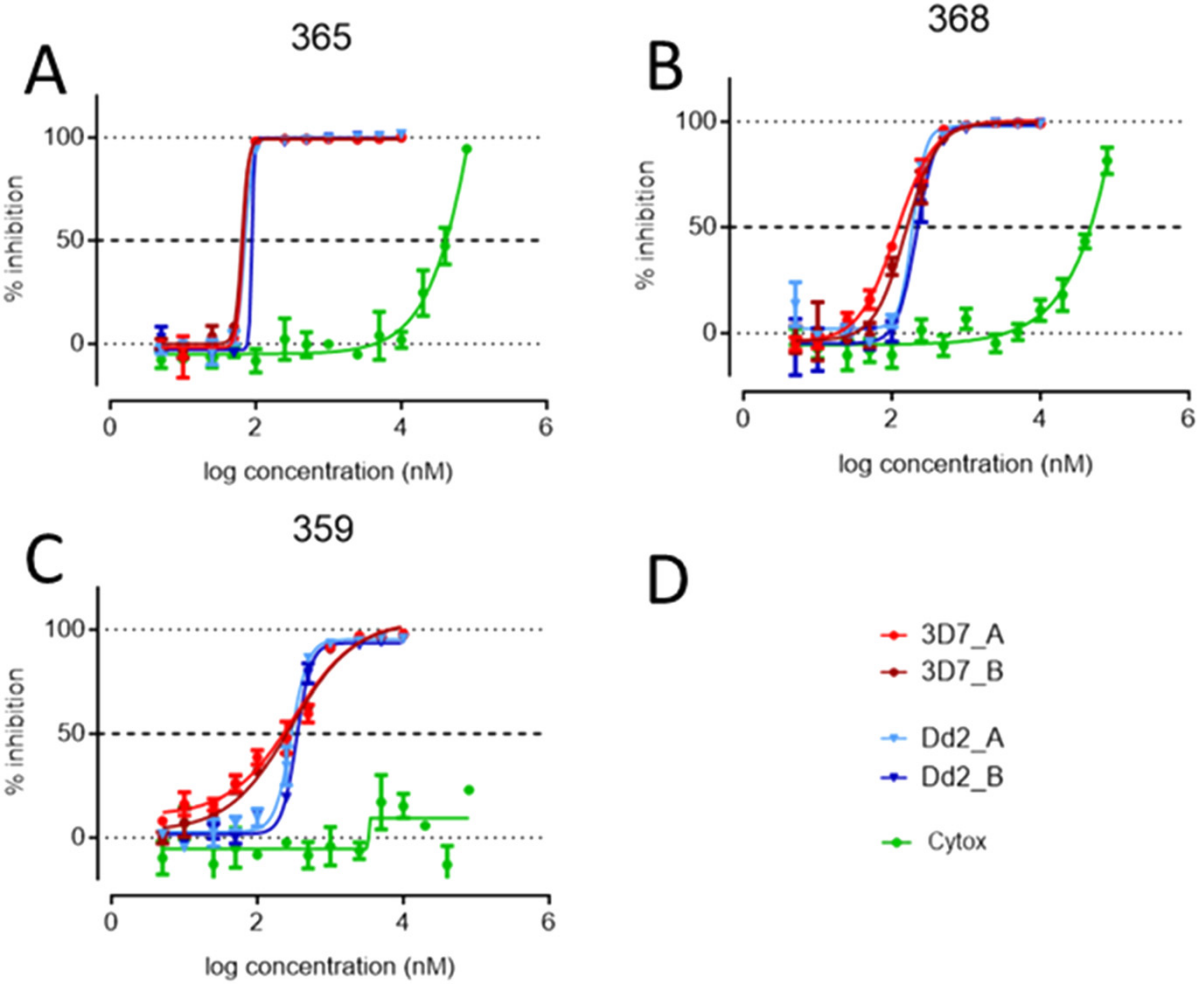
*P. falciparum* (3D7 and Dd2) and HEK293 dose–response curves of S-MGB-365 (Panel A), S-MGB368 (Panel B) and S-MGB-359 (Panel C). Panel D shows the key. Curves labelled as 3D7_A and 3D7_B are for the *P. falciparum* 3D7 strain, two independent experiments in duplicate. Curves labelled as Dd2_A and Dd2_B are for the *P. falciparum* Dd2 strain, two independent experiments in duplicate. Curves labelled as cytox represent the HEK293 assay (duplicate data).

Based on their selectivity indices, and further scrutiny of their dose response curves (Fig. 3), we selected **S-MGB-365** (4a), **S-MGB-368** (4b) and **S-MGB-359** (4c) for further evaluation. We note that the dose response curves for these S-MGBs emphasise the excellent selectivity with respect to HEK293 cytotoxicity.

### 2.3 Assessment of DNA binding

Across other S-MGB drug discovery programmes, double stranded (dsDNA) binding has been assessed using two orthogonal methods, a thermal shift analysis of genomic DNA (gDNA) and native mass spectrometry using short AT-rich dsDNA oligomers.^7,12^**S-MGB-365** (4a), **S-MGB-368** (4b) and **S-MGB-359** (4c) were similarly assessed in this study.

**Fig. 4:**
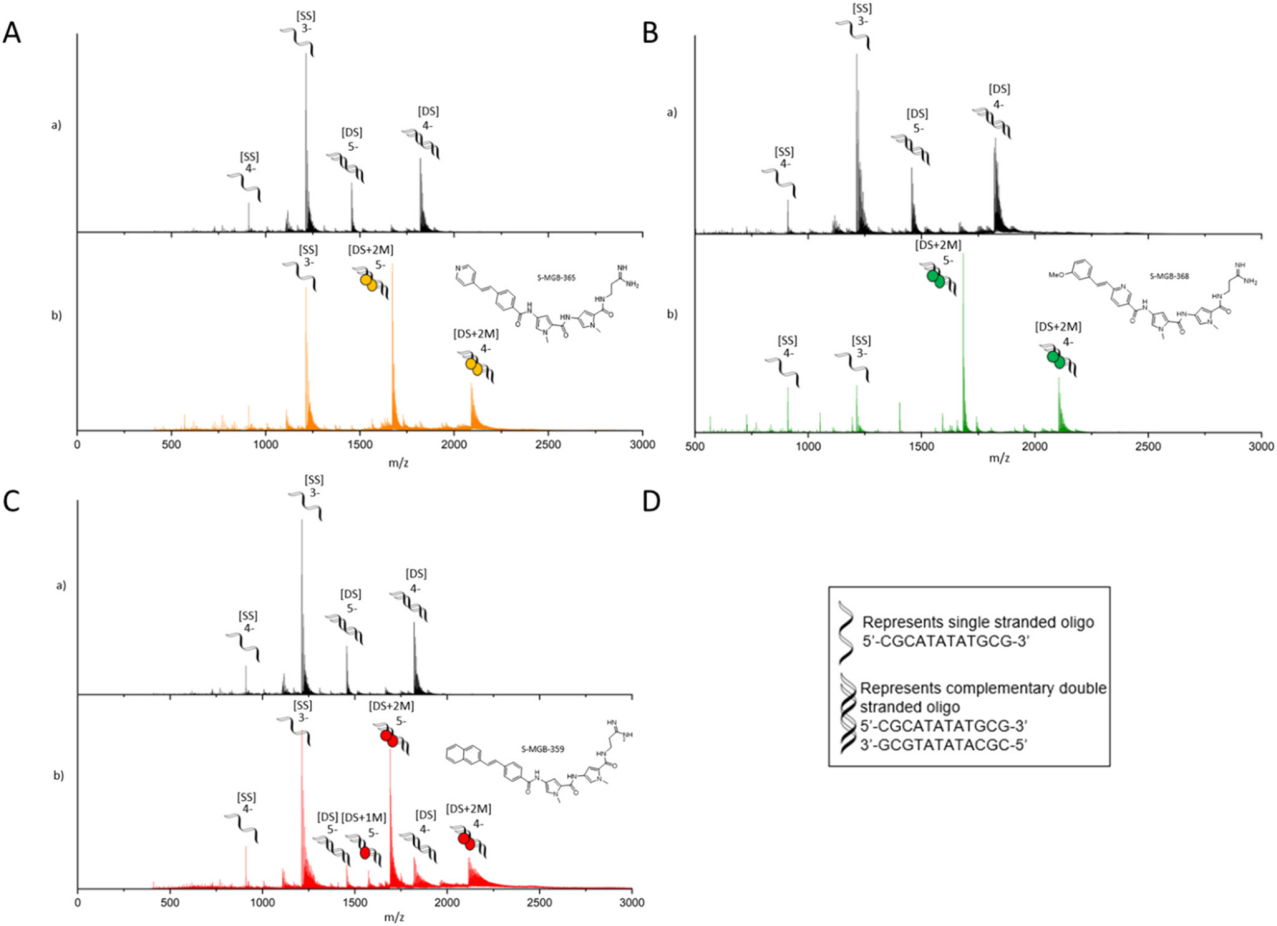
nESI-MS characterisation of S-MGBs binding to double stranded DNA oligo 5′-CGCATATATGCG-3′. 9 μM DNA, (100 μM KCl, 1% DMSO) sprayed from ammonium acetate (150 mM, pH 7) in the absence (a) and presence (b) of 100 μM S-MGBs. For each panel A) S-MGB-365 (4a), B) S-MGB-368 (4b) and C) S-MGB-359 (4c), subpanels show the following: a) single stranded DNA (denoted [SS]) were present in charge states 4- and 3-, and double stranded DNA (denoted [DS]) were present in charge states 5-^−^ and 4-. b) [SS] was present in charge state 3- and 4-. Each [DS] molecule bound 2xS-MGB molecules (denoted [DS+2 M]) and was present in charge states 5-and 4-. For S-MGB-359 (4c), panel C), a small amount of bound [DS] molecules was bound to 1xS-MGB molecule (denoted [DS+1 M]), present in charge state 5-. Expected and measured masses of each species are provided in Tables S2–S4.†

Native mass spectrometry was carried out using a short, self-complementary DNA oligo with an AT-rich binding site, 5′-CGCATATATGCG-3′, which we have used extensively across the S-MGB programme (Fig. 4). For all three S-MGBs, there was conclusive evidence that they bind as a dimer [DS + 2 M], in charge states 5- and 4- (Fig. 3). There was no evidence for **S-MGB-365** (4a) or **S-MGB-368** (4b) bound as a monomer [DS + 1 M]; however, there was a minor peak corresponding to the monomer complex for **S-MGB-359** (4c). For **S-MGB-359** (4c) there was also a small *m*/*z* peak corresponding to the free dsDNA oligo. The presence of the unbound double-stranded DNA, as well as the binding of the monomer S-MGB, suggested that the interaction of **S-MGB-359** (4c) with DNA was weaker than **S-MGB-365** (4a) or **S-MGB-368** (4b), especially due to the high excess of MGB used in this assay.

**Fig. 5:**
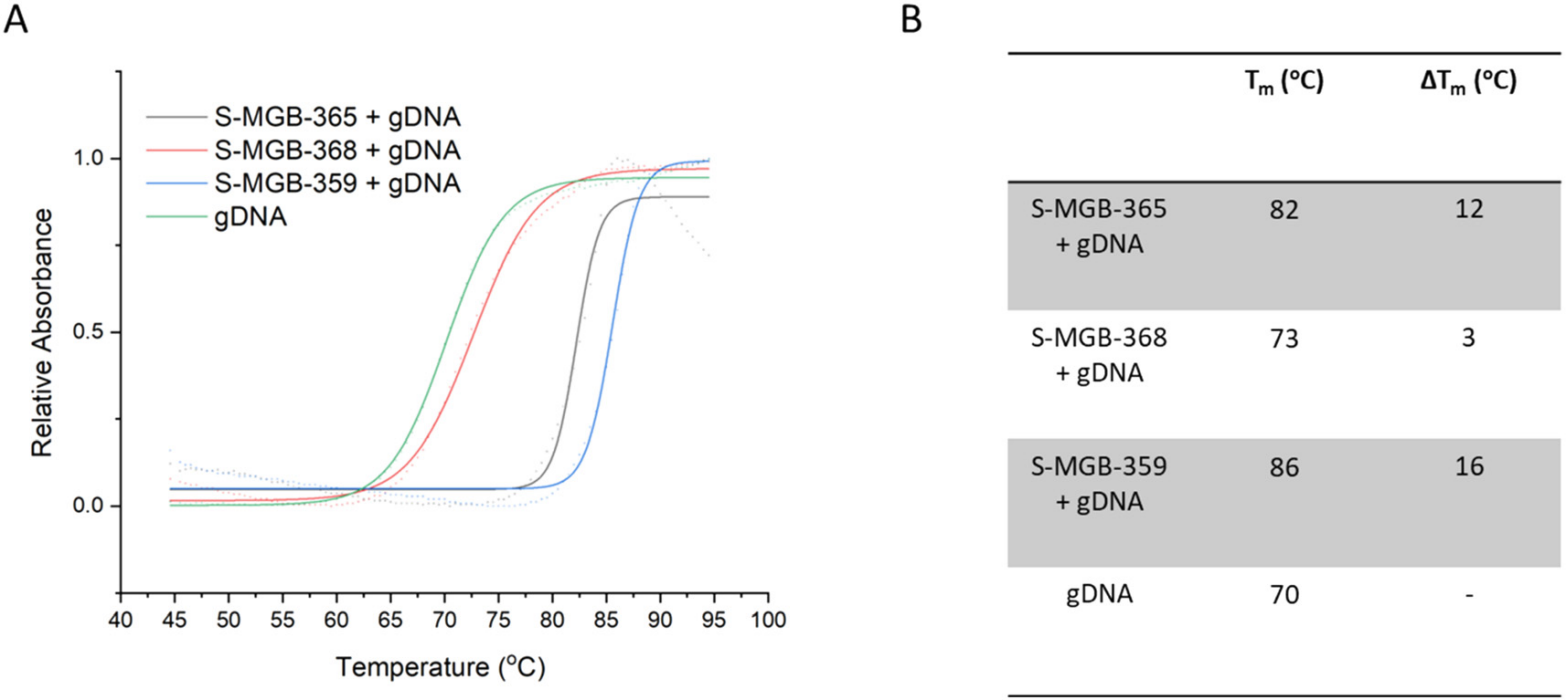
Thermal melt analysis of **S-MGB-365** (4a), **368** (4b), **359** (4c) bound to gDNA including exemplar melt curve from one experimental repeat, fitted with a Boltzmann distribution (A) and computed melting temperatures for *N* = 3 experiments, with all values within ±1 °C (B).

To further probe comparative binding strengths, we also determined the DNA thermal shift of **S-MGB-365** (4a), **S-MGB-368** (4b) and **S-MGB-359** (4c) using a model gDNA extracted from salmon. The Δ*T*_m_s were found to be 12 °C, 3 °C and 16 °C for **S-MGB-365** (4a), **S-MGB-368** (4b) and **S-MGB-359** (4c), respectively (Fig. 5). Considering these values alongside the *P. falciparum* IC_50_ values, a correlation between DNA binding strength and potency was not evident. This is consistent with a dominating contribution from intracellular accumulation over target engagement governing potency.

## 3. Conclusion

Previous, and ongoing, studies into S-MGB structure–activity relationships (SAR) suggest that those bearing an amidine tail group, rather than a tertiary amine, are less cytotoxic due to reduced intracellular accumulation.^5,7,10^ However, we had not investigated the antiplasmodial activity of S-MGBs bearing an amidine tail group in our previous study.^9^ Indeed, **S-MGB-169**, identified in that previous study, possessed a morpholinoethyl tail group reminiscent of **MGB-BP-3**, the antibacterial compound that has completed Phase IIa clinical trials for the treatment of *C. difficile*.^5^

In this study 25 S-MGBs, including 5 that were previously unpublished, have been synthesised and evaluated in *in vitro* asexual *P. falciparum* assays against *P. falciparum* 3D7 and Dd2, and HEK293 mammalian cells. This set of S-MGBs included a variety of contemporary structures, including those bearing an amidine tail group. Similar to our previous study on the antiplasmodial activity of S-MGBs, we found the S-MGBs in this study to range in their potency and selectivity, allowing for an assessment of SAR.

**S-MGB-365** (4a), **S-MGB-368** (4b) and **S-MGB-359** (4c) have been identified as both potent (∼100 nM) and selective compounds and importantly no cross-resistance observed with the strains tested. Notably, the compounds of most interest, based on a combination of potency and selectivity, **S-MGB-365** (4a), **S-MGB-368** (4b) and **S-MGB-359** (4c) all contain an amidine tail group. However, there does not appear to be a correlation between amidine tail group and potency against *P. falciparum* alone, suggesting that this moiety is important in driving low cytotoxicity towards mammalian cells. We also note that the behaviour of S-MGB-359 against HEK293 cells suggests it to be substantially less cytotoxic than the other compounds assessed (Panel C, Fig. 3). This different behaviour may be associated with its likely amphiphilic nature, given that head group in this molecule is a hydrophobic naphthyl moiety.

It has been well established in recent literature that similar S-MGBs, including those with amidine tail groups, interact with DNA by forming 2 : 1 complexes at suitable AT-rich binding sites.^5,10^**S-MGB-365** (4a), **S-MGB-368** (4b) and **S-MGB-359** (4c) have been shown to adhere to this principle, strongly suggesting that the mechanism of action of antiplasmodial S-MGBs involves DNA binding, and this is in line with antibacterial and antitrypanosomal S-MGBs.^7,8^

However, DNA binding strength alone is not sufficient to explain potency trends. Despite having the highest DNA thermal melting temperature of the three compounds of interest, **S-MGB-359** (4c) has the lowest potency. This observation suggests that molecular properties that contribute to accumulation within pathogen cells are also a key component to potency, in addition to DNA binding. Indeed, this is consistent with other S-MGB drug discovery programmes, and explains the low potency towards Gram-negative bacterial pathogens despite strong binding to Gram-negative bacterial DNA.^5,7,10^

In conclusion, **S-MGB-365** (4a), **S-MGB-368** (4b) and **S-MGB-359** (4c) have been identified as promising compounds from which to continue drug discovery efforts towards new antiplasmodial agents due to their potency, selectivity, and lack of cross-resistance mechanisms.

## 4. Experimental

### 4.1 *In vitro* antiplasmodial assay

The antiplasmodial *in vitro* assay was performed as previously described.^13^ In brief, *P. falciparum* parasites (3D7 and Dd2 strains) were grown in RPMI-1640 medium supplemented with 25 mM HEPES, 5% AB human male serum, 2.5 mg mL^−1^ Albumax II, and 0.37 mM hypoxanthine. Parasites were subjected to two rounds of sorbitol synchronization prior to compound addition. Ring stage parasites were incubated with compounds in 384-well imaging CellCarrier microplates, for 72 h at 37 °C, 90% N_2_, 5% CO_2_, 5% O_2_, parasites stained with 4′,6-diamidino-2-phenylindole (DAPI) and imaged using an Opera QEHS microplate confocal imaging system (PerkinElmer). Images were analysed as previously described,^13^ and raw data normalized using the in-plate positive and negative controls to obtain percent inhibition, used to calculate IC_50_ values, through a four-parameter logistic curve fitting in Prism (GraphPad).

### 4.2 HEK293 assay

Cell viability was assessed using a minor modification of the protocol previously described.^14^ Human embryonic kidney cells (HEK293) were maintained in Dulbecco's modified Eagle's medium supplemented with 10% foetal bovine serum. HEK293 cells were incubated with compounds in TC-treated 384-well plates (Greiner) for 72 h at 37 °C, 5% CO_2_, then media removed from the wells and replaced with an equal volume of 44 μM resazurin. After 5–6 h incubation as described above, the total fluorescence (excitation/emission: 530/595 nm) was measured using an Envision plate reader (PerkinElmer).

### 4.3 UV-vis DNA thermal melting experiments

Salmon genomic DNA (gDNA; D1626, Sigma-Aldrich) at 1 mg mL^−1^ in 1 mM phosphate buffer (pH 7.4) containing 0.27 mM KCl and 13.7 mM NaCl (P4417, Sigma-Aldrich) was annealed at 90 °C for 10 min and left to cool to room temperature. S-MGBs at 10 mM in DMSO were diluted with the same phosphate buffer to yield a single sample with 10 μM S-MGB and 0.02 mg mL^−1^ gDNA in 1 mM phosphate buffer containing 0.27 mM KCl and 13.7 mM NaCl. Control samples containing only S-MGB or gDNA were prepared, respectively. Samples were melted at a rate of 0.5 °C min^−1^ from 45 °C to 90 °C with spectra recorded at 260 nm on a UV-1900 UV-vis spectrophotometer fitted with a Peltier temperature controller (Shimadzhu) using LabSolutions (Tm Analysis) software. The melting temperatures (Tms) of the S-MGB:DNA complexes were determined by fitting a sigmoidal function using a Boltzmann distribution in OriginPro.

### 4.4 Native mass spectrometry

#### DNA sample preparation

DNA oligonucleotide sequence 5′-CGCATATATGCG-3′ was purchased in lyophilized form from Alpha DNA, Canada) and used without further purification, purity assessed by NMR. 100 μM stock solutions of DNA were prepared with 150 mM ammonium acetate solution (Fisher Scientific, Loughborough, Leicestershire, UK) and 2 mM potassium chloride solution (Fisher Scientific, Loughborough, Leicestershire, UK). This solution was annealed at 90 degrees for 10 minutes and allowed to cool to room temperature. 10 mM S-MGB stock in 100% DMSO (Sigma-Aldrich, St. Louis, MO, USA) were diluted to 1 mM S-MGB solution with 150 mM ammonium acetate. Final samples were prepared from this solution to yield final concentrations of 9 μM DNA, 100 μM KCl, and 100 μM s-MGB, 1% DMSO. DNA solutions containing no S-MGB included 1% DMSO and were used as controls.

#### Mass spectrometry measurements

Native mass spectrometry experiments were carried out on a Synapt G2Si instrument (Waters, Manchester, UK) with a nanoelectrospray ionization source (nESI). Mass calibration was performed by a separate infusion of NaI cluster ions. Solutions were ionized from a thin-walled borosilicate glass capillary (i.d. 0.78 mm, o.d. 1.0 mm, Sutter Instrument Co., Novato, CA, USA) pulled in-house to nESI tip with a Flaming/Brown micropipette puller (Sutter Instrument Co., Novato, CA, USA). A negative potential in range of 1.0–1.2 kV was applied to the solution *via* a thin platinum wire (diameter 0.125 mm, Goodfellow, Huntingdon, UK). The following non-default instrument parameters were used for the DNA: S-MGB-241 complex: capillary voltage 1.4 kV, sample cone voltage 100 V, source offset 110 V, source temperature 40 °C, trap collision energy 3.0 (V), trap gas 3 mL min^−1^. For DNA with no MGB present, the following parameters were changed: capillary voltage 1.0 kV, sample cone voltage 80 V, source offset 95 V and trap gas 4.0 mL min^−1^. Data were processed using Masslynx V4.2 and OriginPro 2021, and figures were produced using Chemdraw.

### 4.5 Compound synthesis

Details of compound synthesis and characterisation are found in the ESI.†

## Author contributions

FJS and AIK carried out the chemical synthesis. VMA oversaw the *Plasmodium falciparum* studies and cytotoxicity testing. VMA and TRG analysed the *Plasmodium* and cytotoxicity data. LMCM performed the native mass spectrometry experiments. MP carried out the DNA thermal melt experiments. Further intellectual contributions to the project and its management were made by LMCM, MP, TRG, RB, CJS, VMA and FJS. All authors contributed to writing their respective parts of the manuscript. Executive manuscript preparation, drafting, and management were carried out by MP, CJS, RB, VMA and FJS.

## Conflicts of interest

The authors declare the following competing financial interest(s): MP, LMCM, AIK, CJS and FJS are part of revenue sharing agreements with their University relating to the Strathclyde minor groove binder project. Additionally, CJS and FJS have financial interests through shares in the company, Rostra Therapeutics. All other authors: none to declare.

## Supplementary Material

MD-016-D4MD00619D-s001

## Data Availability

The data supporting this article have been included as part of the ESI.†
