# Supplementary material for: S-MGBs bearing amidine tail groups are potent, selective antiplasmodial agents
Source: RSC Med Chem. 2024 Oct 16;16(1):412–9. doi: 10.1039/d4md00619d (PMC11528320; doi:10.1039/d4md00619d)
Supplement: MD-016-D4MD00619D-s001 [file MD-016-D4MD00619D-s001.pdf]

Supplementary Information (SI) for RSC Medicinal Chemistry.
This journal is © The Royal Society of Chemistry 2024

S-MGBs bearing amidine tail groups are potent, selective antiplasmodial agents: Supplementary Information

Marina PERIETEANU,[1] Tayner RODRIGUEZ GARZON,[2,3] Leah M. C. MCGEE,[1] Abedawn I. KHALAF, [1] Colin J. SUCKLING,[1] Rebecca BEVERIDGE,[1] Vicky M. AVERY[2,3], and Fraser J. SCOTT[1*]

[1]Department of Pure and Applied Chemistry, University of Strathclyde, Glasgow UK

[2]Discovery Biology, Centre for Cellular Phenomics, Griffith University, Nathan, Queensland 4111, Australia

[3] School of Environment & Sciences, Griffith University, Nathan, Queensland, 4111, Australia

*Email: fraser.j.scott@strath.ac.uk

Table of Contents

1. S-MGB Structures, page 2

2. Synthesis Details, page 7

 2.1 General Methods, page 7

 2.2 Synthesis Details for Headgroups, page 7

 2.3 General Procedure for Novel S-MGBs, page 8

 2.4 Characterisation Data for Novel S-MGBs, page 8

3. *In Vitro* Asexual *P. Falciparum* Assays Against 3D7 and Dd2 For S-MGBs, page 16

4. *In Vitro* Cytotoxicity Assessment Against HEK293 For S-MGBs, page 20

5. *In Vitro* Asexual *P. Falciparum* Assays Against 3D7 and Dd2, And Cytotoxicity Assessment Against HEK293 For Control Compounds, page 23

6. Native Mass Spectrometry, page 24

1. S-MGB Structures

S-MGB ID	Structure
**365**	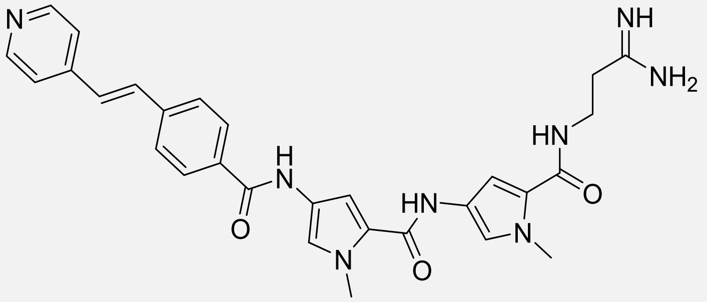
**368**	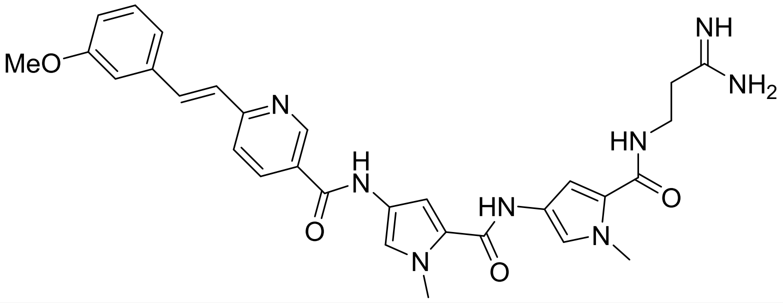
**359**	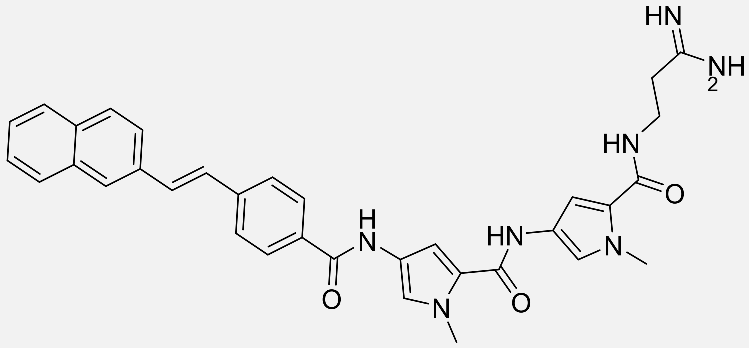
**337**[b]	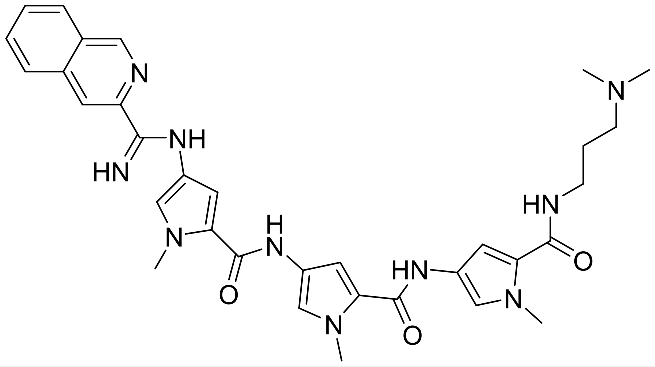
**188**[c]	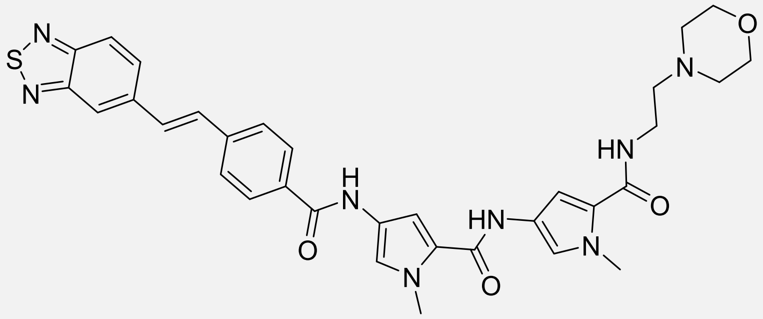

380[d]

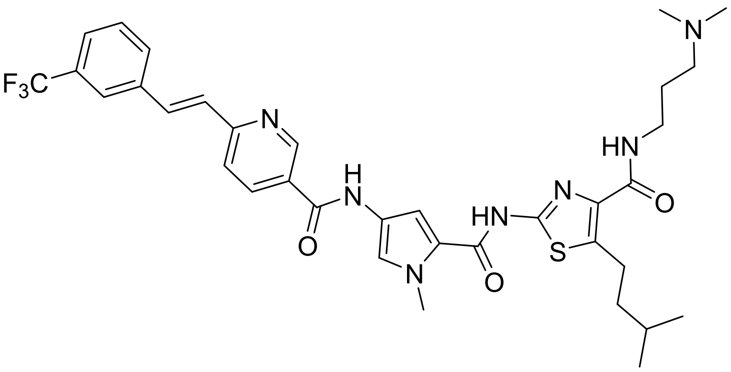

388

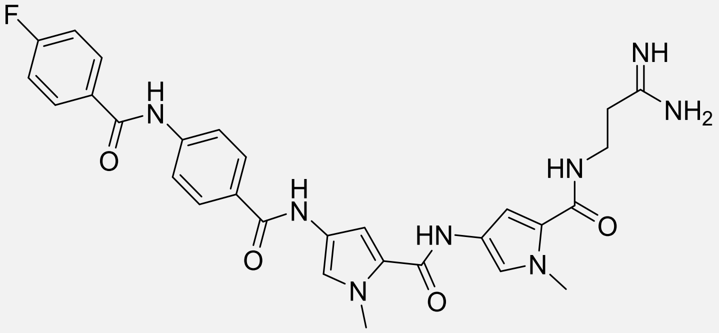

131[e]

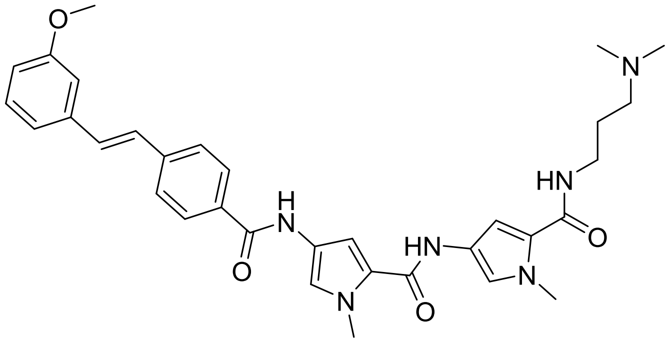

248[a]

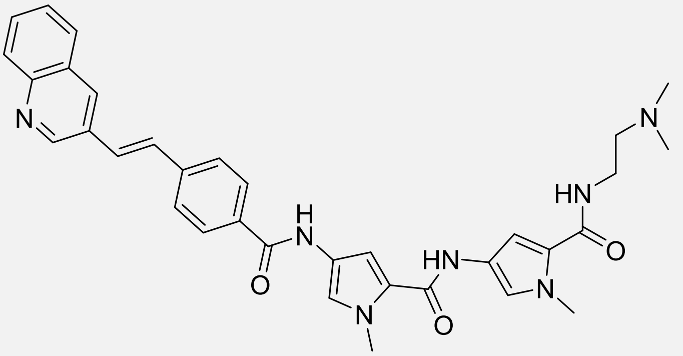

247[a]

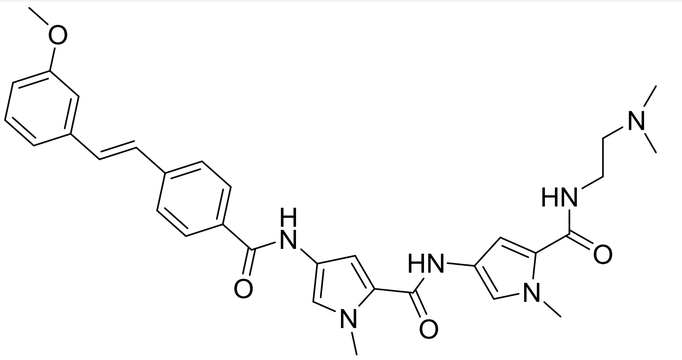

246[a]

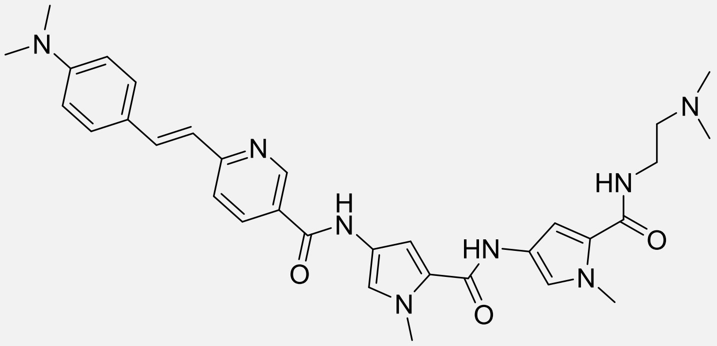

379[d]

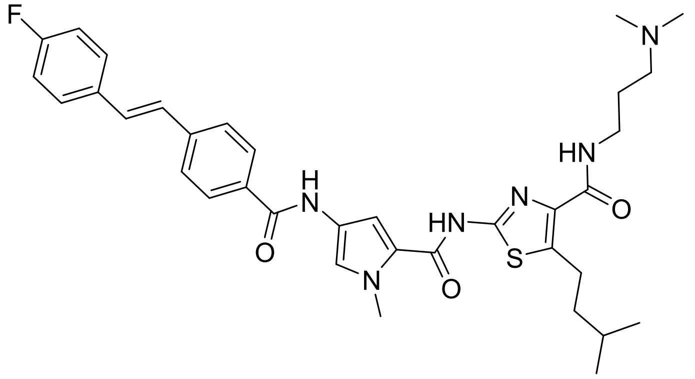

386[d]

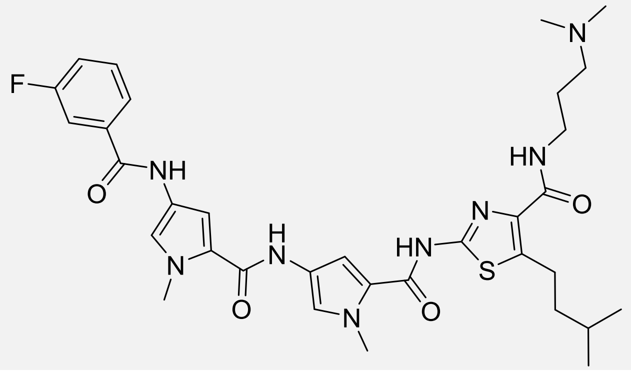

378[d]

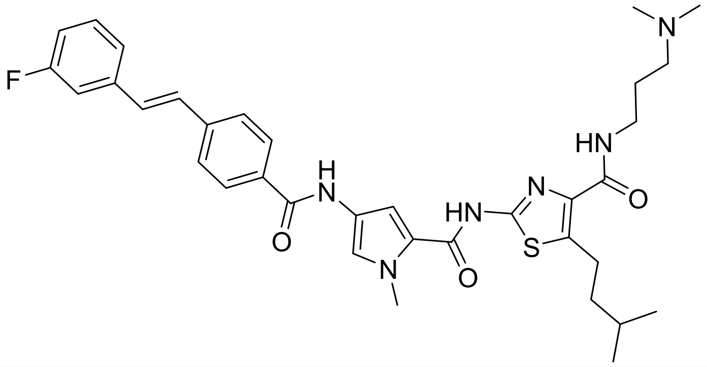

376[d]

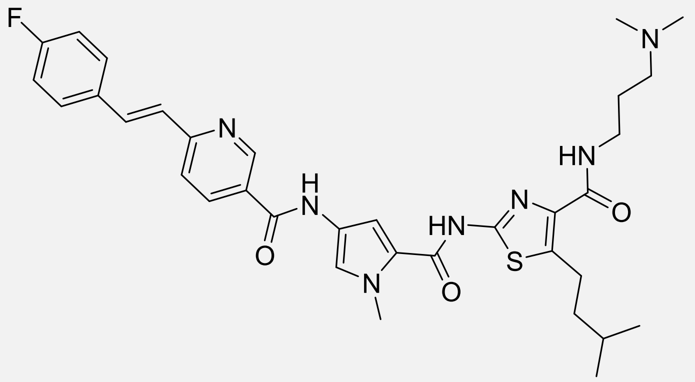

377[d]

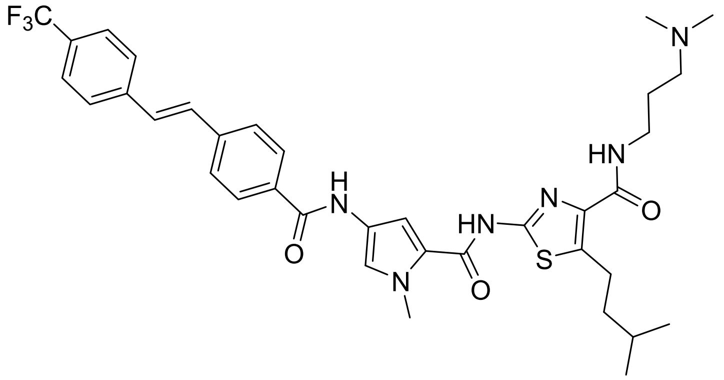

361

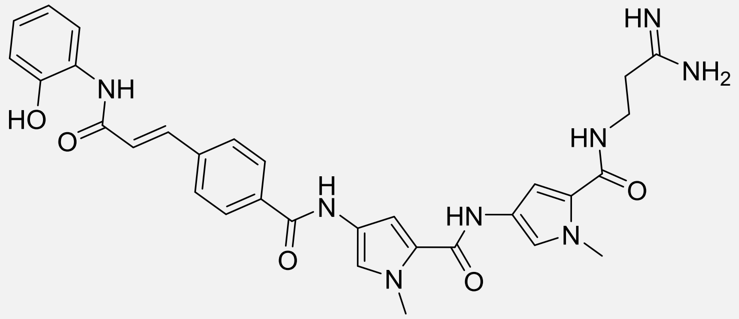

367[f]

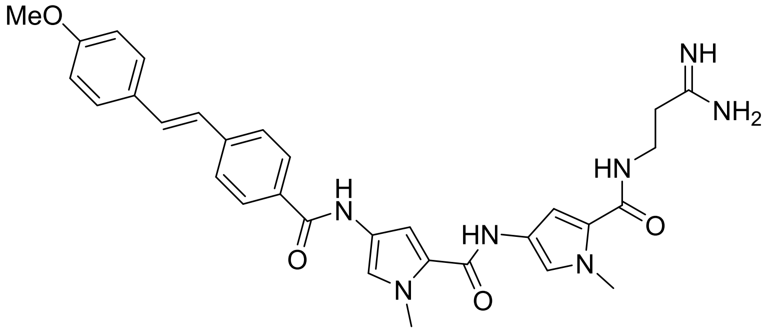

390[f]

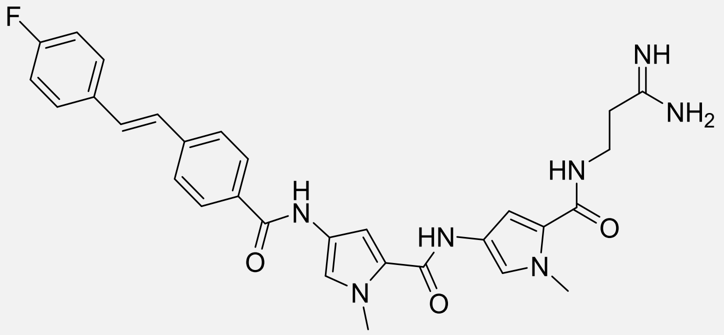

192[g]

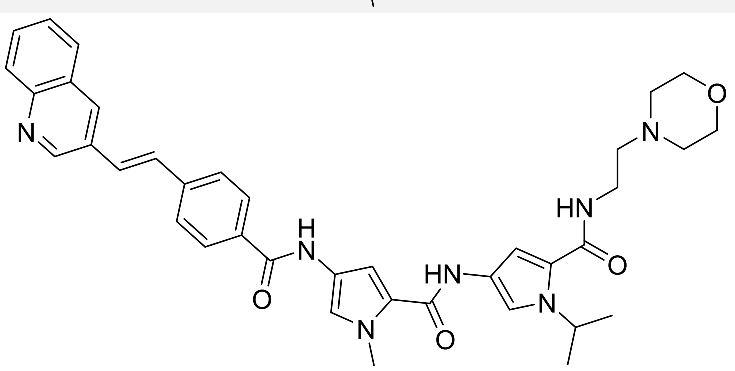

176[c]

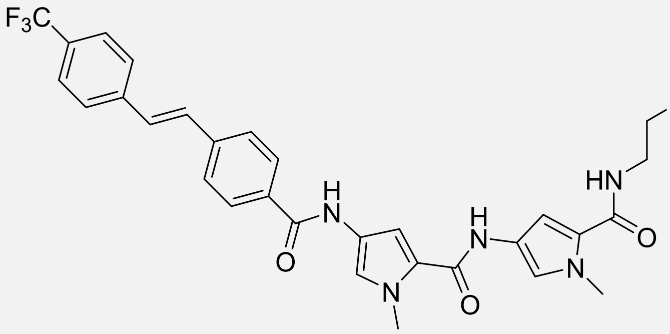

391[f]

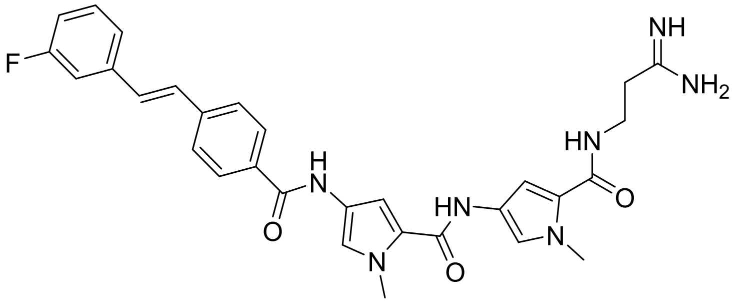

330[b]

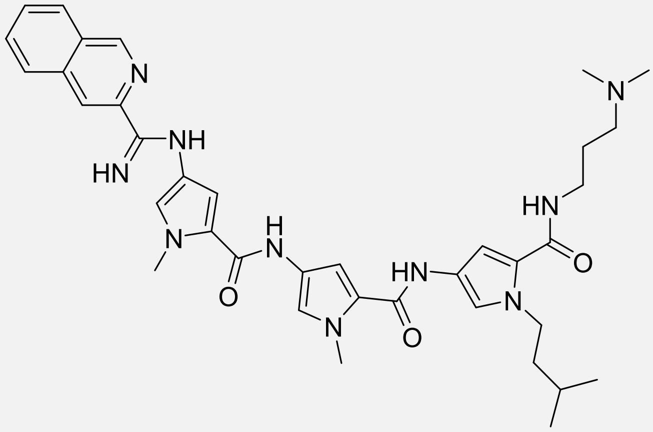

389[f]

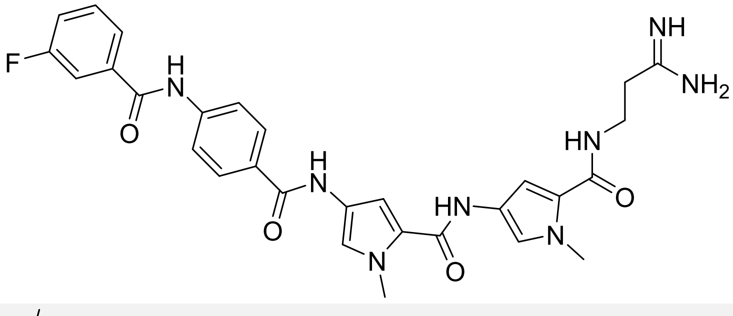

245[c]

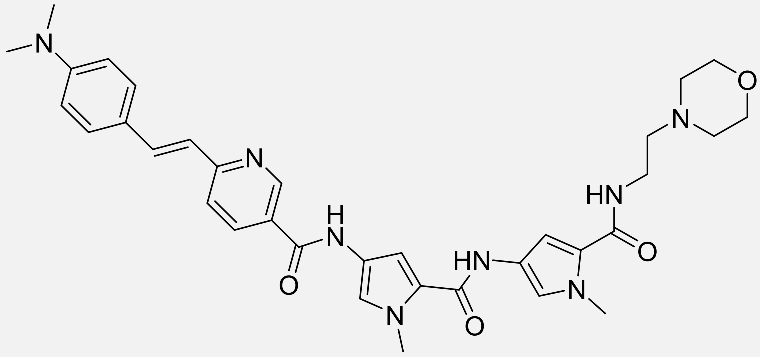

Table S1. S-MGB Structures. Compounds isolated as TFA salts. Subscripts indicate location synthesis details of these compounds: [a]https://doi.org/10.1021/acs.jmedchem.8b01847 [b]https://doi.org/10.1016/j.ejmech.2017.05.039 [c]https://doi.org/10.1016/j.ejmech.2016.03.064 [d]https://doi.org/10.1039/C9MD00268E [e]https://doi.org/10.1021/jm070831g [f]manuscript submitted [g]https://doi.org/10.1016/j.ejmech.2011.08.035

2. Synthesis Details

2.1 General Methods

Reagents and Solvents. Used as supplied from Aldrich unless dry DCM or dry THF. These were provided by standard operating procedures for InnovativeTechnology Solvent Purification System.

Solvent Removal. This was carried out by evaporation using a rotary evaporator at reduced pressure (*ca* 20 mmHg) unless otherwise stated.

Thin Layer Chromatography. TLC was carried out using pre-coated silica plates (Alugram® Sil G/UV_{254}). Visualisation of TLC plates was achieved by UV (254 nm).

Melting Point. Reichert hot stage melting point apparatus and are uncorrected.

NMR. Bruker Spectrospin 400 MHz or 500 MHz operating frequency for ^{1}H NMR nuclei. δ quoted in ppm and measured relative to residual proton from the solvent. Coupling constants, *J,* are given in Hz. ^{13}C NMR run at 125 MHz and measured relative to the solvent.

IR. Mattson 1000 FTIR spectrometer (Unicam Analytical Systems). Spectrum software. Frequencies are quoted in cm^{-1}.

HR-MS-FAB. Recorded on a Jeol JMS-700 M STATION high resolution magnetic sector spectrometer.

Column Chromatography. Silica gel mesh size 230-400 (40-60µm).

Preparative HPLC. Instrument Setup: Waters 1525 Binary HPLC Pump, Waters 717plus Autosampler, Waters 2487 Dual λ Absorbance Detector. LC Conditions: Column, Luna 5µ C18(2) 100A; Dimensions, 60 × 21.1 mm; Injection Volume, 100 µL; Solvent Flow Rate, 6 mL/min; Detection wavelength, 254 nm; solvent A, water (with 0.1% TFA) and solvent B, MeCN (with 0.1% TFA), with run-to-to changes in gradient to achieve separation.

2.2 Synthesis Details for Headgroups 3a-e

3a As per https://doi.org/10.1021/jm070831g
3c As per https://doi.org/10.1021/jm070831g
3d As per https://doi.org/10.1016/j.ejmech.2020.112411
3e As per https://doi.org/10.1021/jm070831g

Procedure for the Preparation of 3b

Methyl 6-[(*E*)-2-(3-methoxyphenyl)ethenyl]nicotinate

3-Methoxybenzaldehyde (210 mg, 1.52 mmol), methyl 6-methylnicotinate (230 mg, 1.52 mmol), acetic anhydride (310 mg, 3.04 mmol) and catalytic amount of zinc chloride were heated at 140^{0}C with stirring for 12 h. Ethyl acetate and brine were added to the cooled reaction mixture and the product was extracted. The organic layer was collected, dried ($MgSO_4$) and the solvent removed under reduced pressure. The crude product was applied to a silica gel column and the product was eluted with ethyl acetate/n-hexane 1:1. Fractions containing the required product were collected and the solvents removed under reduced pressure to give the desired material as a yellow solid (87 mg, 21%), mp170- 173°C.

IR (cm^{-1}).1717, 1606, 1591, 1511, 1433, 1290, 1254, 1175, 1111, 1020, 844, 818, 760, 734

^{1}H NMR (500 MHz, DMSO-d_6) δ (ppm) 9.04 (1H, d, *J* = 2.0Hz), 8.25 (1H, dd, *J* = 2.2Hz, *J* = 8.2Hz), 7.81 (1H, d, *J* = 16.0Hz), 7.67-7.62 (3H, m), 7.28 (1H, d, *J* = 16.0Hz), 7.00 (2H, d,

J = 8.8Hz), 3.88 (3H, s), 3.80 (3H, s).

HRMS m/z calculated for $C_{16}H_{16}O_3N$ 270.1130 $[M+H]^+$, found 270.1127 $[M+H]^+$

6-[(*E*)-2-(3-Methoxyphenyl)ethenyl]nicotinic acid, 3b

Methyl 6-[(*E*)-2-(3-methoxyphenyl)ethenyl]nicotinate (80 mg, 0.297 mmol) was dissolved in methanol (5 mL) to which sodium hydroxide solution (145 mg) in water (10 mL) was added. The reaction mixture was heated under reflux for 3 h. Methanol was removed under reduced pressure and the remaining solution was cooled to 0^{0}C. Hydrochloric acid (conc.) was added dropwise with vigorous stirring until pH 4. The yellow solid material was collected by filtration, washed with water and dried under reduced pressure at 50°C to give the required material (59 mg, 78%), mp 230-233°C

IR (cm^{-1}) 1717, 1681, 1635, 1595, 1513, 1292, 1250, 1173, 1023, 825

^{1}H NMR (500 MHz, DMSO-d_6) δ (ppm) 9.03 (1H, d, *J* = 2.0Hz), 8.29 (1H, dd, *J* = 2.1Hz, *J* = 8.2Hz), 7.90-7.65 (4H, m), 7.29 (1H, d, *J* = 16.0Hz), 7.01 (2H, m), 3.80 (3H, s)

HRMS m/z calculated for $C_{15}H_{14}O_3N$ 256.0974 $[M+H]^+$, found 256.0972 $[M+H]^+$

2.3 General Procedure for Novel S-MGBs

Nitro tail group dimer **1**, (prepared as per https://doi.org/10.1021/acs.jmedchem.8b01847) (50 mg, 0.123 mmol) was dissolved in methanol (10 mL) to which Pd/C-10% (40 mg) was added at 0°C under nitrogen with stirring. The reaction mixture was hydrogenated for 4h at room temperature and atmospheric pressure. The catalyst was removed over Kieselguhr and the solvent was removed under reduced pressure. The amine so formed was taken forward directly without further purification. Amide coupling of the amine with the appropriate head group acid (**3a-3e**) (0.123 mmol) was carried out in the presence of HBTU (0.17 mmol) in DMF (2 mL) and triethylamine (0.17 mmol), for 18 hours at room temperature. The reaction mixture was then purified directly by HPLC, followed by freeze drying, to afford the final S-MGB compounds as their TFA salts.

2.4 Characterisation Details for Novel S-MGBs

S-MGB-365, 4a, (E)-4-(4-((5-((5-((3-amino-3-iminiopropyl)carbamoyl)-1-methyl-1H-pyrrol-3-yl)carbamoyl)-1-methyl-1H-pyrrol-3-yl)carbamoyl)styryl)pyridin-1-ium di-trifluoroacetate

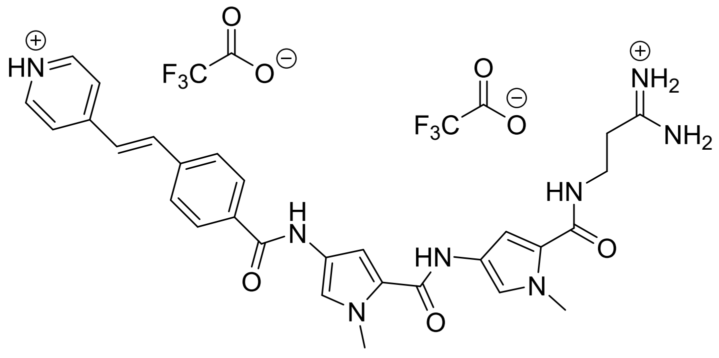

Yellow solid (17% yield) with no distinct melting point.

IR: 721, 748, 801, 841, 974, 1126, 1196, 1269, 1310, 1404, 1437, 1468, 1516, 1570, 1622, 1676 cm^{-1}

^{1}H NMR (DMSO-d_6): 10.39(1H, s), 9.96(1H, s), 8.91(2H, s), 8.69(2H, s), 8.49(2H, s), 8.21(1H, t, J = 5.4Hz), 8.03(2H, d, J = 8.3Hz), 7.85-7.76(5H, m), 7.51(1H, d, J = 16.3Hz),7.35(1H, d, J = 1.5Hz), 7.19(1H, d, J = 1.5Hz), 7.13(1H, d, J = 1.5Hz), 6.98(1H, d, J = 1.5Hz), 3.89(3H, s), 3.83(3H, s), 3.53(2H, q, J = 6.5 Hz), 2.63(2H, t, J = 6.5Hz).

HRMS: Found: M+2/2: 270.1294 Calculated for $C_{29}H_{32}O_3N_8$ 270.1293

1H-NMR Spectrum:

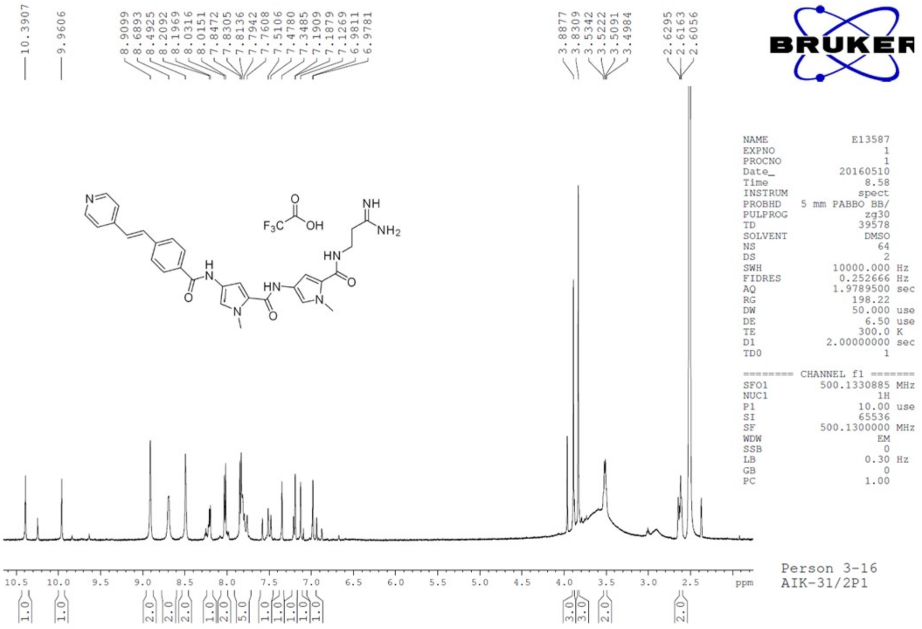

IR Spectrum:

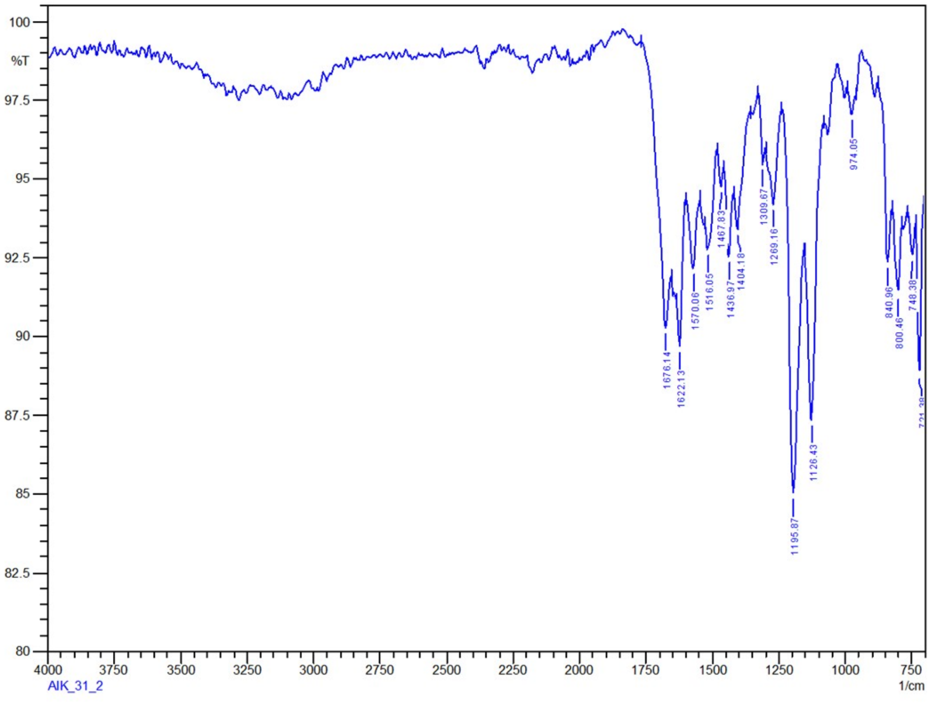

S-MGB-368, 4b, (E)-5-((5-((5-((3-amino-3-iminiopropyl)carbamoyl)-1-methyl-1H-pyrrol-3-yl)carbamoyl)-1-methyl-1H-pyrrol-3-yl)carbamoyl)-2-(3-methoxystyryl)pyridin-1-ium di-trifluoroacetate

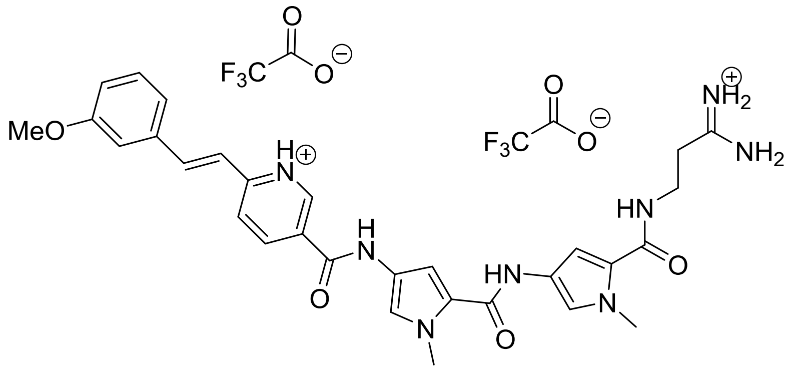

White solid (23% yield) with no distinct melting point.

IR: 720, 775, 799, 833, 961, 1005, 1045, 1126, 1182, 1254, 1290, 1404, 1433, 1462, 1528, 1578, 1630, 1659 cm^{-1}

^{1}H NMR (DMSO-d$_6$): 10.51(1H, s), 9.97(1H, s), 9.10(1H, d, J = 2.0Hz), 8.91(2H, s), 8.48(2H, s), 8.31(1H, dd, J = 2.5Hz & J = 8.0Hz), 8.22(1H, t, J = 5.5Hz), 7.79(1H, d, J = 16.0Hz, double bond), 7.71(1H, d, J = 8.5Hz), 7.45(1H, d, J = 16.0 Hz, double bond), 7.37-7.34(4H, m), 7.29(1H, d, J = 6.5Hz), 7.19(1H, d, J = 1.5Hz), 7.121-7.118(1H, d, J = 1.5Hz), 6.97(1H, d, J = 1.5Hz), 6.95(1H, dd, J = 1.5Hz & J = 8.0Hz), 3.89(3H, s), 3.83(6H, s), 3.54(2H, q, J = 6.5Hz), 2.63(2H, t, J = 6.5Hz).

HRMS: Found: 569.2622 Calculated for $C_{30}H_{33}O_4N_8$ 569.2619

1H-NMR Spectrum:

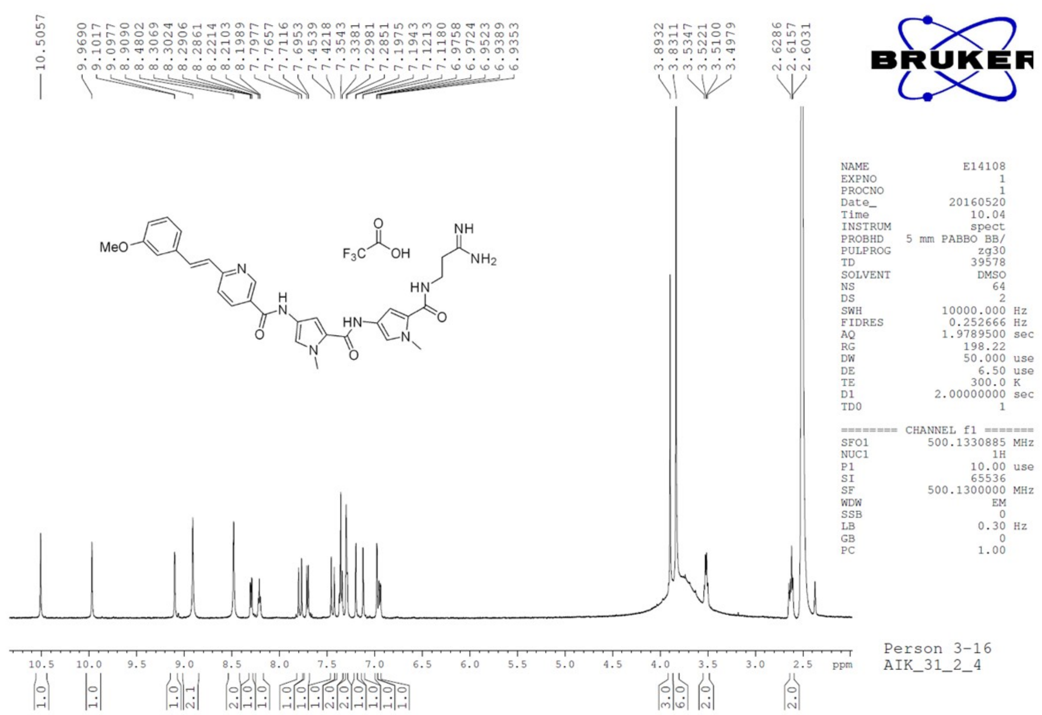

IR Spectrum:

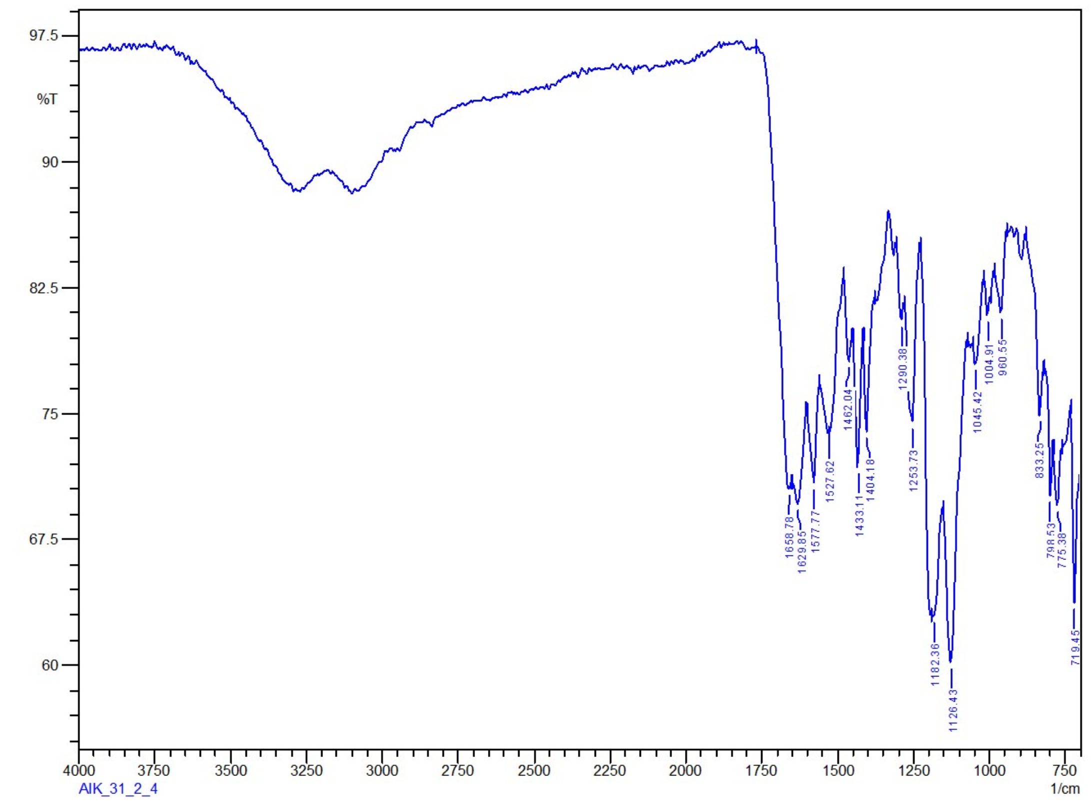

S-MGB-359, 4c, (E)-1-amino-3-(1-methyl-4-(1-methyl-4-(4-(2-(naphthalen-2-yl)vinyl)benzamido)-1H-pyrrole-2-carboxamido)-1H-pyrrole-2-carboxamido)propan-1-iminium trifluoroacetate

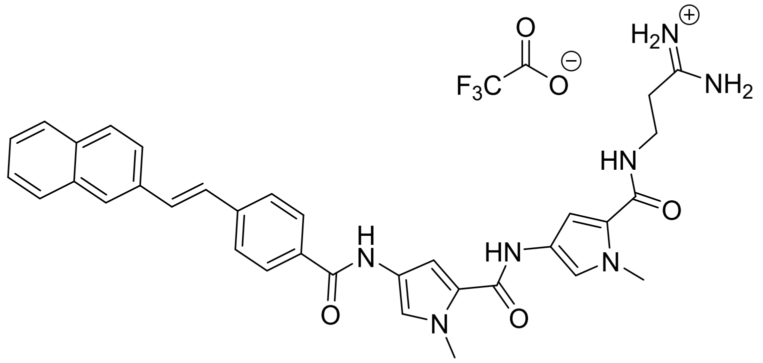

White solid (32% yield) with no distinct melting point.

IR: 720, 743, 801, 833, 895, 959, 1013, 1063, 1128, 1182, 1200, 1265, 1402, 1433, 1464, 1528, 1580, 1636, 1670 cm^{-1}

^{1}H NMR (DMSO-d_6): 10.35(1H, s), 9.96(1H, s), 8.91(2H, s), 8.49(2H, s), 8.22(1H, t, J = 5.7Hz), 8.08(1H, s), 8.01(2H, d, 8.3Hz), 7.97-7.92(4H, m), 7.82(2H, d, J = 8.3Hz), 7.61-7.49(4H, m), 7.35(1H, d, J = 1.4Hz), 7.20(1H, d, J = 1.4Hz), 7.13(1H, d, J = 1.4Hz), 6.98(1H, d, J = 1.4Hz), 3.89(3H, s), 3.83(3H, s), 3.54(2H, q, J = 6.3Hz), 2.63(2H, t, J = 6.3Hz).

HRMS: Found: 588.2714 Calculated for $C_{34}H_{34}O_3N_7$ 588.2718

1H-NMR Spectrum:

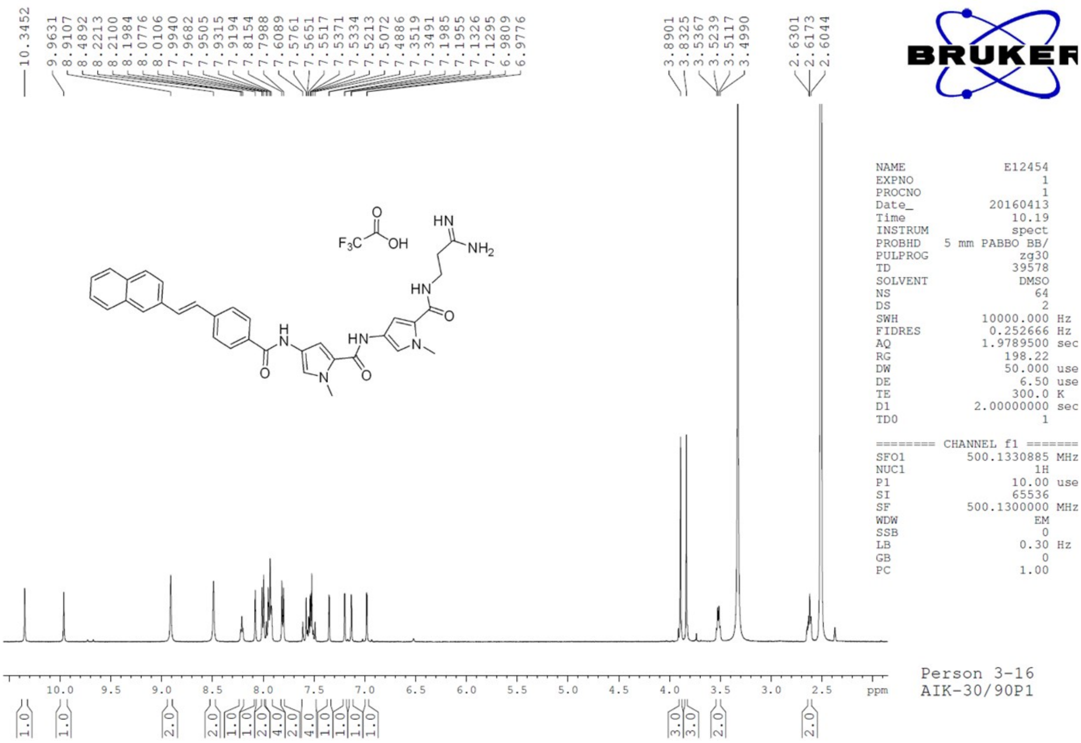

10.3452
9.9631
8.9107
8.4892
8.2213
8.2100
8.1984
8.0776
8.0106
7.9940
7.9682
7.9505
7.9315
7.9194
7.8154
7.7988
7.6089
7.5761
7.5651
7.5517
7.5371
7.5334
7.5213
7.5072
7.4886
7.3519
7.3491
7.1985
7.1955
7.1326
7.1295
6.9809
6.9776
3.8901
3.8325
3.5367
3.5239
3.5117
3.4990
2.6301
2.6173
2.6044
BRUKER
NAME E12454
EXPNO 1
PROCNO 1
Date_ 20160413
Time 10.19
INSTRUM spect
PROBHD 5 mm PABBO BB/
PULPROG zg30
TD 39578
SOLVENT DMSO
NS 64
DS 2
SWH 10000.000 Hz
FIDRES 0.252666 Hz
AQ 1.9789500 sec
RG 198.22
DW 50.000 use
DE 6.50 use
TE 300.0 K
D1 2.00000000 sec
TD0 1
======== CHANNEL f1 =======
SFO1 500.1330885 MHz
NUC1 1H
P1 10.00 use
SI 65536
SF 500.1300000 MHz
WDW EM
SSB 0
LB 0.30 Hz
GB 0
PC 1.00
Person 3-16
AIK-30/90P1
ppm

IR Spectrum:

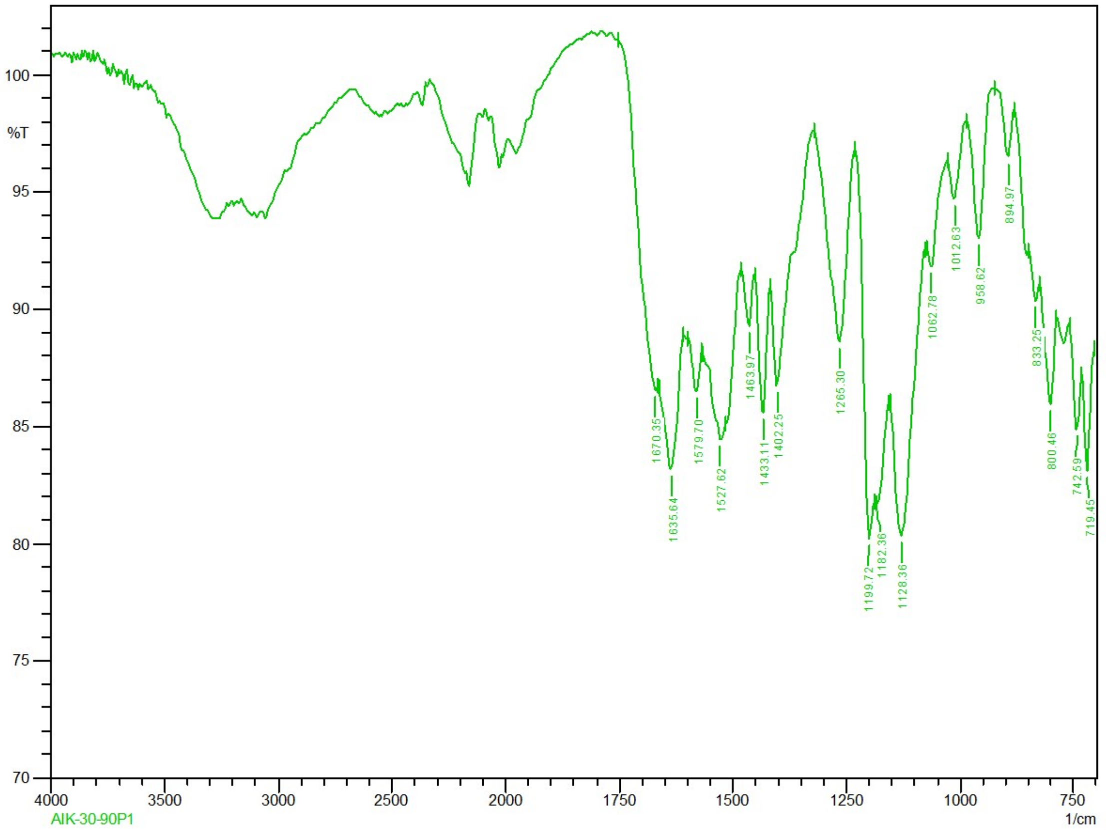

%T
1670.35
1635.64
1579.70
1527.62
1463.97
1433.11
1402.25
1265.30
1199.72
1182.36
1128.36
1062.78
1012.63
958.62
894.97
833.25
800.46
742.59
719.45
AIK-30-90P1
1/cm

S-MGB-388, 4d, 1-amino-3-(4-(4-(4-(4-fluorobenzamido)benzamido)-1-methyl-1H-pyrrole-2-carboxamido)-1-methyl-1H-pyrrole-2-carboxamido)propan-1-iminium trifluoroacetate

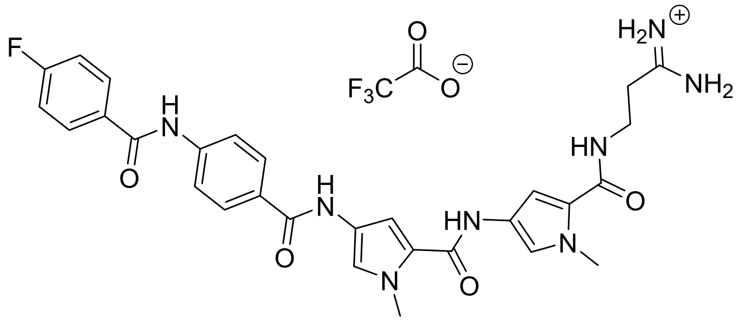

White solid (18% yield) with no distinct melting point.

IR: 719, 758, 798, 848, 898, 1010, 1062, 1126, 1161, 1182, 1199, 1234, 1263, 1290, 1325, 1404, 1435, 1465, 1502, 1527, 1577, 1600, 1629, 1639, ~3050, ~3300 cm^{-1}

^{1}H NMR (DMSO-d_6): 10.50(1H, s), 10.24(1H, s), 9.95(1H, s), 8.90(2H, s), 8.52(2H, s), 8.22(1H, t, J = 5.7Hz), 8.05-8.09(2H, m), 7.97 (2H, d, 8.8Hz), 7.91(2H, d, 8.8Hz), 7.38-7.43(2H, m), 7.32(1H, d, J = 1.8Hz), 7.19(1H, d, J = 1.8Hz), 7.10(1H, d, J = 1.8Hz), 6.97(1H, d, J = 1.8Hz), 3.88(3H, s), 3.83(3H, s), 3.51(2H, q, J = 6.3Hz), 2.61(2H, t, J = 6.3Hz).

HRMS: Found: 573.2375 calculated for $C_{29}H_{30}O_4N_8F$ 573.2369

1H-NMR Spectrum:

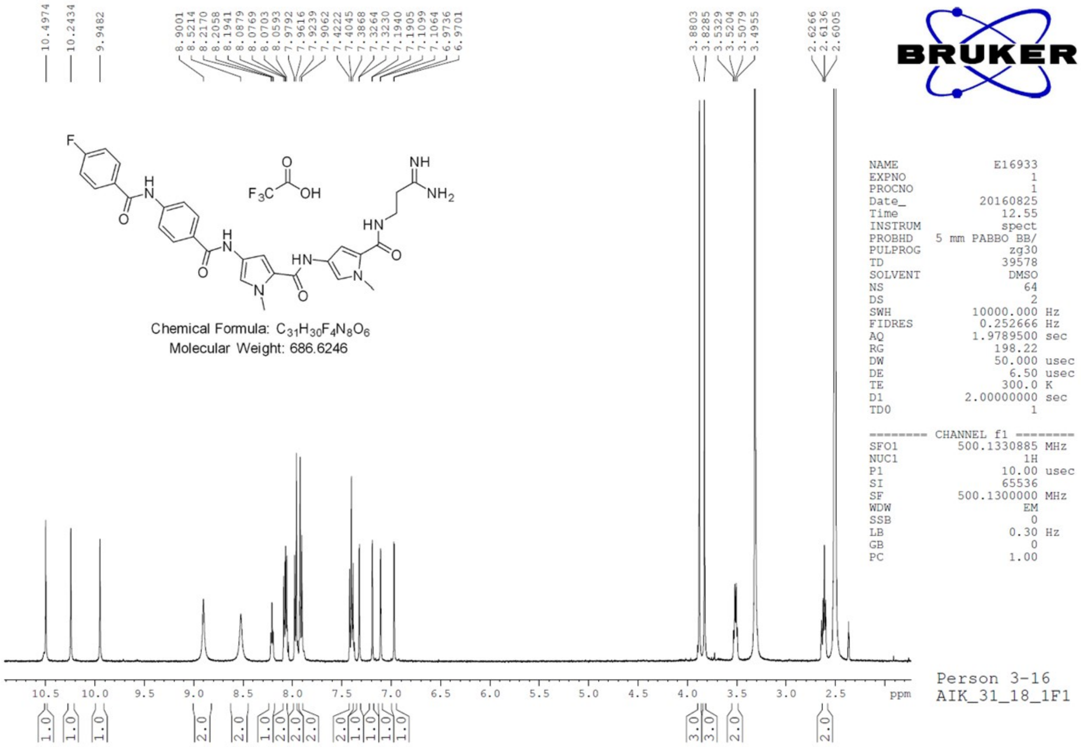

IR Spectrum:

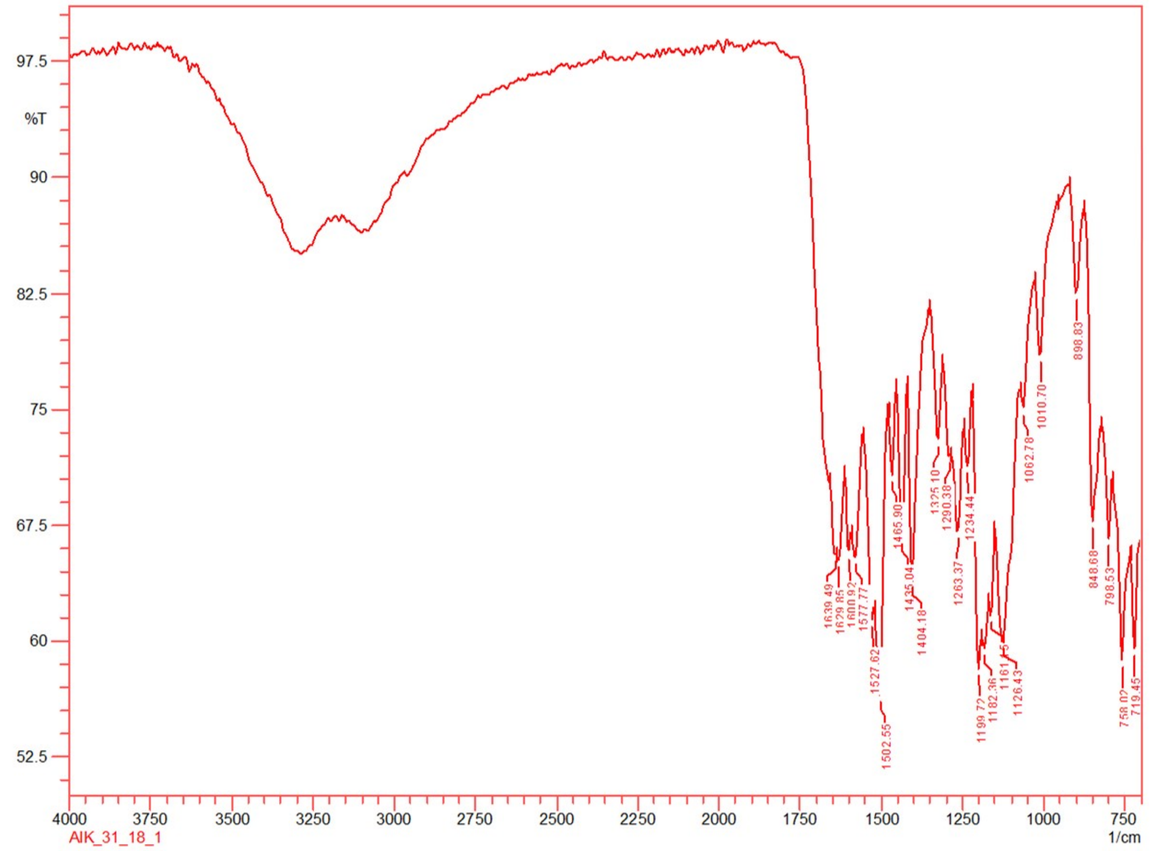

S-MGB-361, 4e, (E)-1-amino-3-(4-(4-(4-(3-((2-hydroxyphenyl)amino)-3-oxoprop-1-en-1-yl)benzamido)-1-methyl-1H-pyrrole-2-carboxamido)-1-methyl-1H-pyrrole-2-carboxamido)propan-1-iminium trifluoroacetate

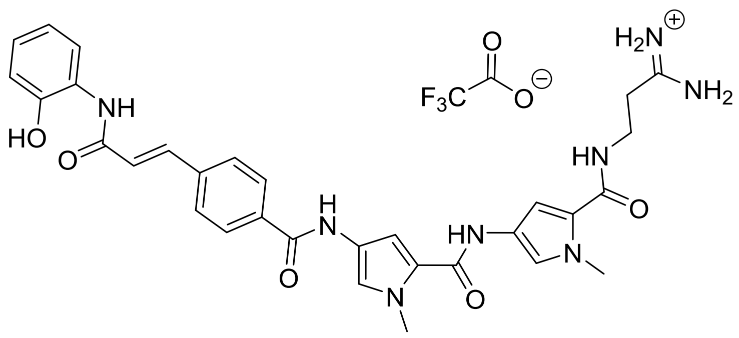

Yellow solid (11% yield) with no distinct melting point.

IR: 721, 748, 799, 837, 976, 999, 1126, 1182, 1200, 1271, 1350, 1404, 1435, 1451, 1526, 1578, 1632, 1655, 1670 cm^{-1}

^1H NMR (DMSO-d_6): 10.45(1H, s), 10.04(1H, s), 10.02(1H, s), 9.56(1H, s), 8.96(2H, s), 8.54(2H, s), 8.27(1H, t, 5.6Hz), 8.08(3H, m), 7.84(2H, d, 8.2Hz), 7.70(1H, d, J = 15.6Hz), 7.40(1H, t, J = 1.3Hz), 7.39(1H, d, J = 9.2Hz), 7.35(1H, d, J = 1.4Hz), 7.25(1H, d, J = 1.4Hz), 7.05(2H, m), 6.97(1H, d, J = 7.0Hz), 6.89(1H, t, J = 7.9Hz), 3.94(3H, s), 3.88(3H, s), 3.59(2H, q, J = 6.5Hz), 2.68(2H, t, J = 6.5Hz).

HRMS: Found: 597.2567 Calculated for $C_{31}H_{33}O_5N_8$ 597.2579

1H-NMR Spectrum:

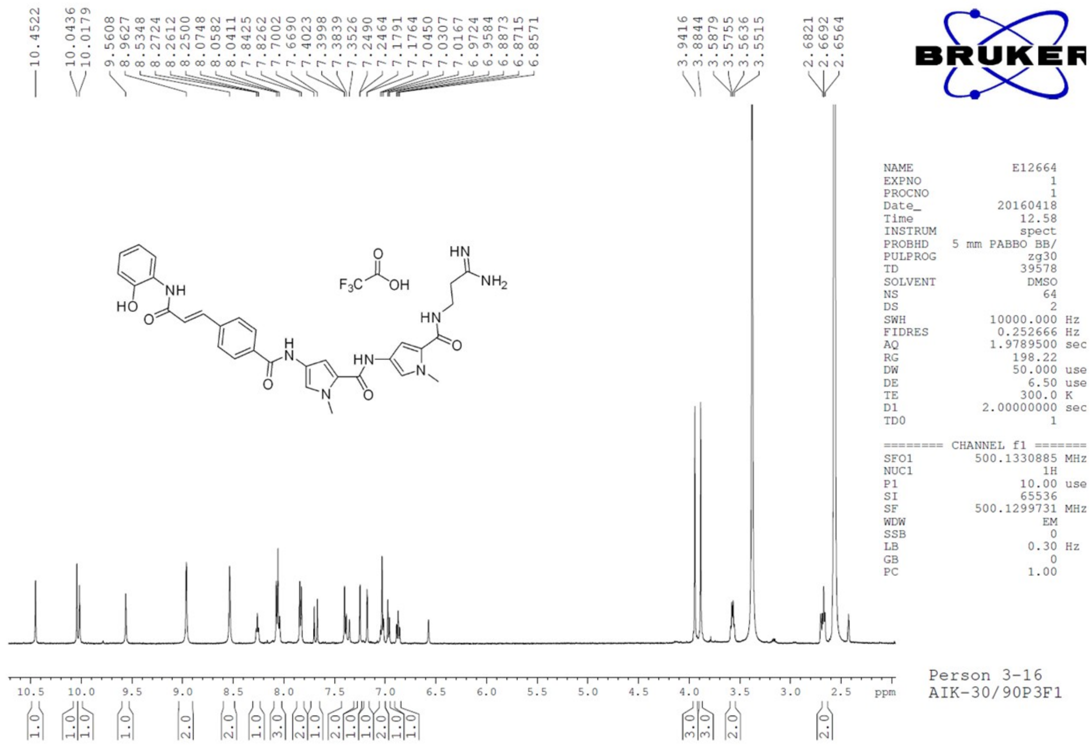

IR Spectrum:

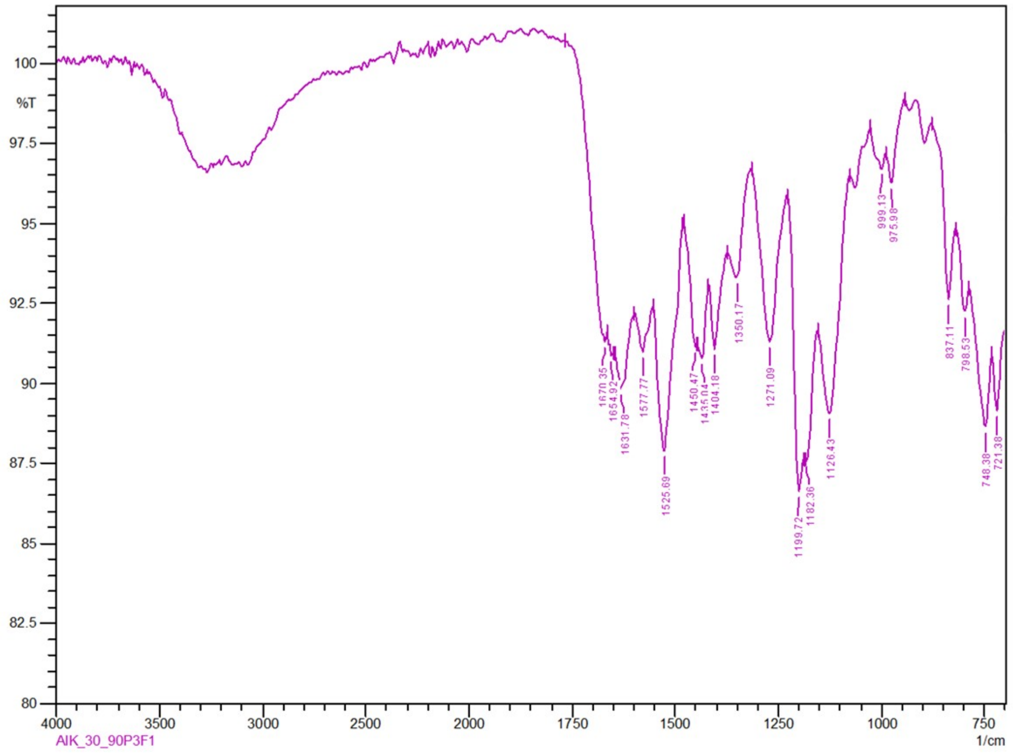

3. *In Vitro* Asexual *P. Falciparum* Assays Against 3D7 and Dd2 For S-MGBs

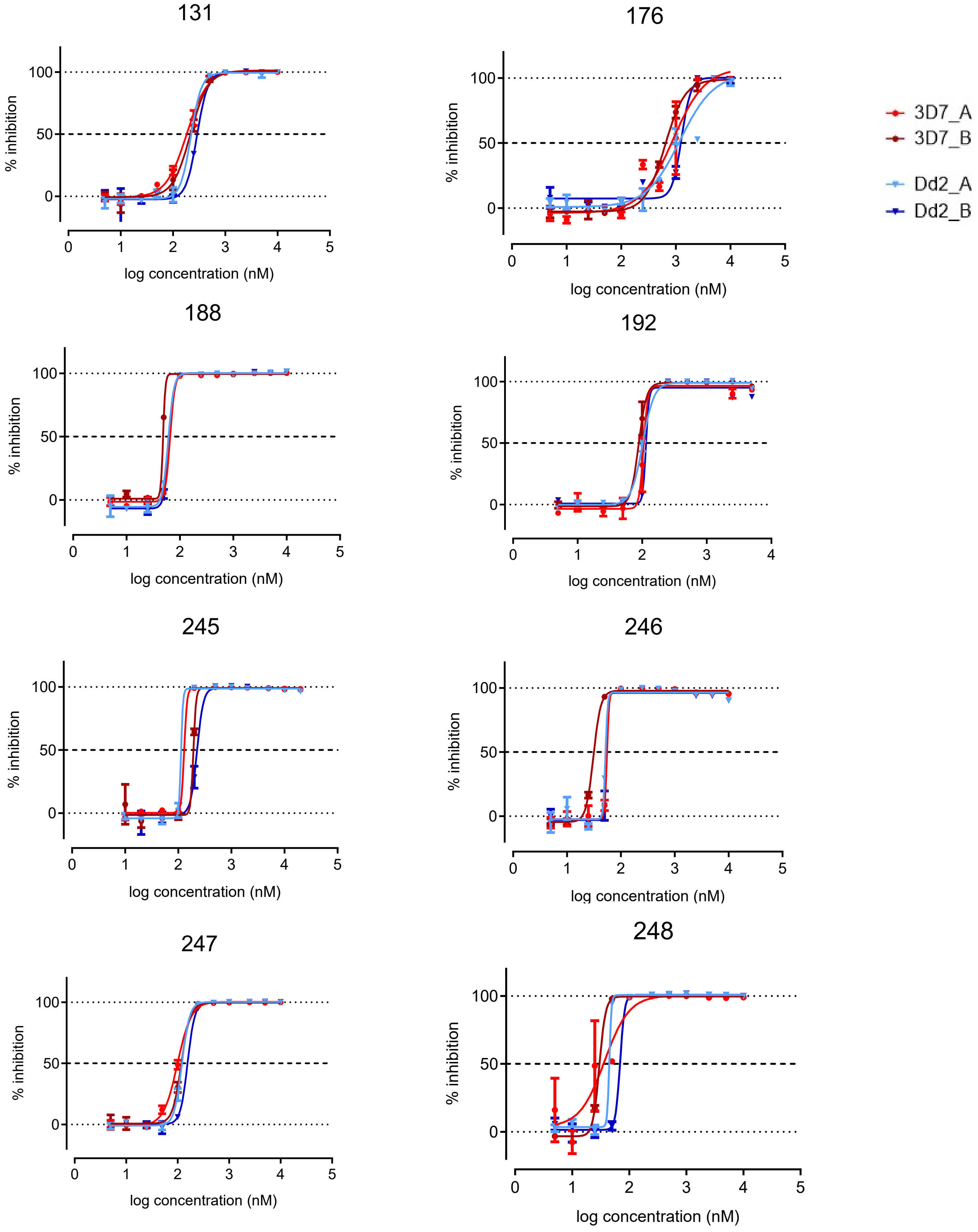

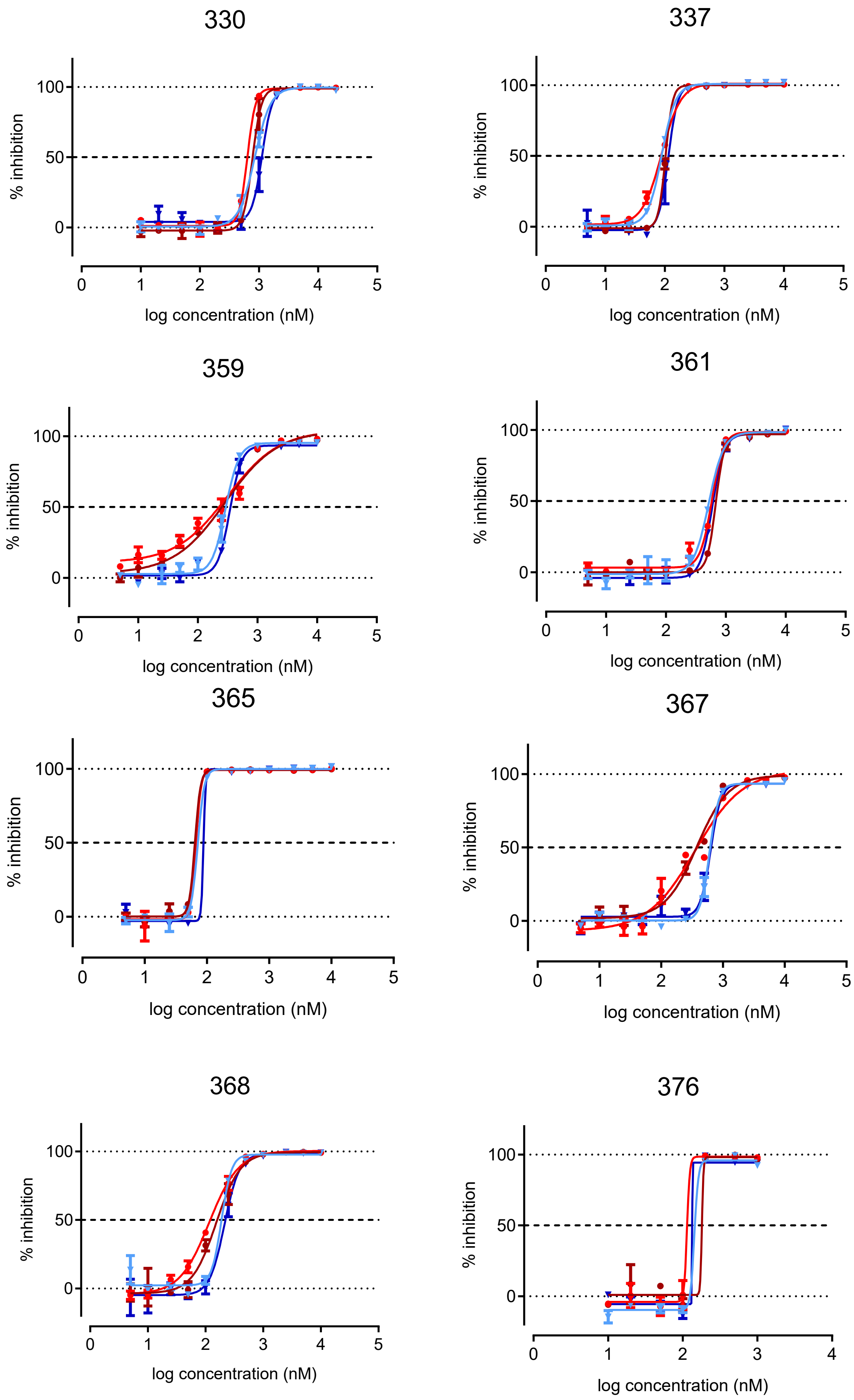
330
337
359
361
365
367
368
376
% inhibition
log concentration (nM)
100
50
0
0
1
2
3
4
5

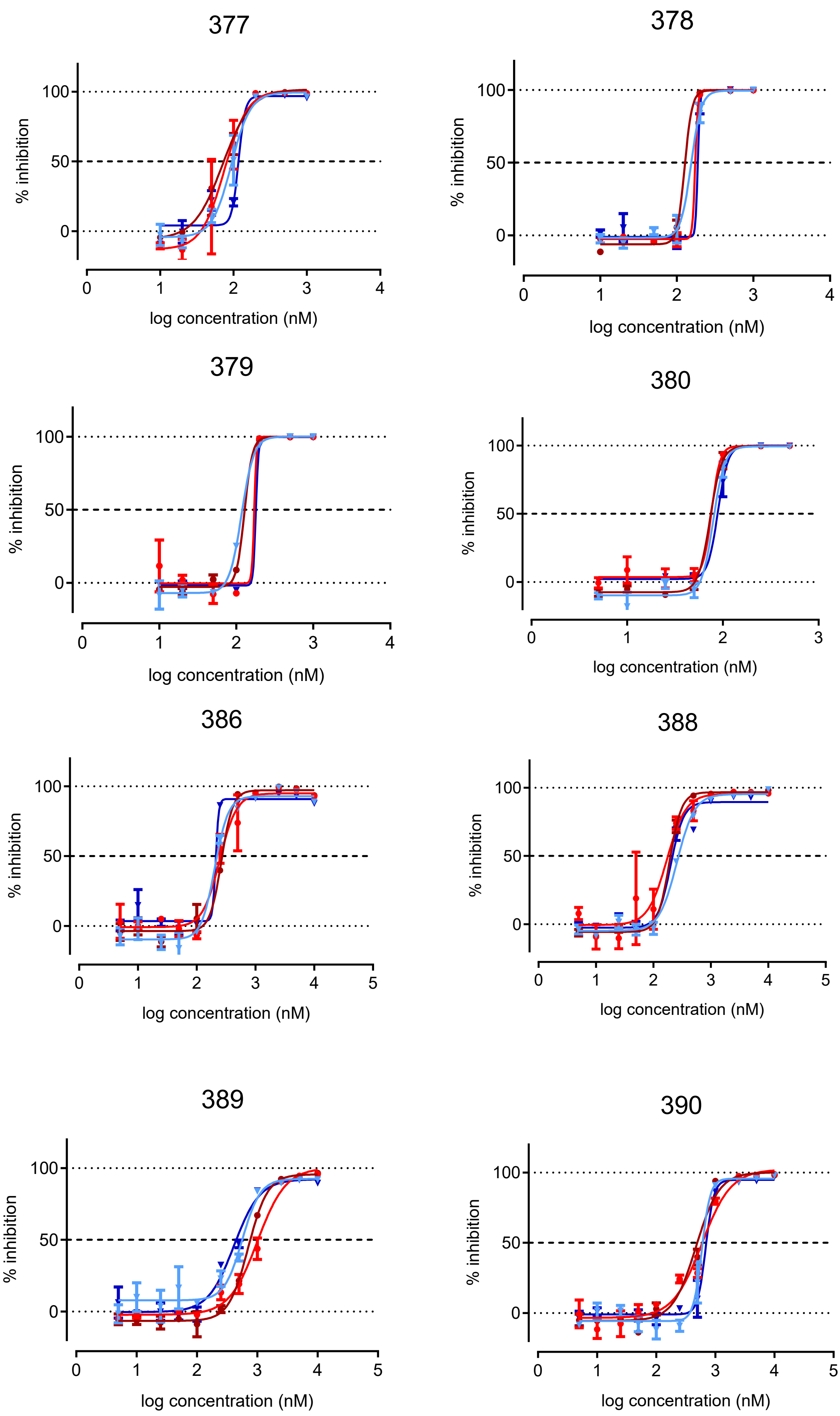
377
378
379
380
386
388
389
390
% inhibition
log concentration (nM)
100
50
0
0
1
2
3
4
5

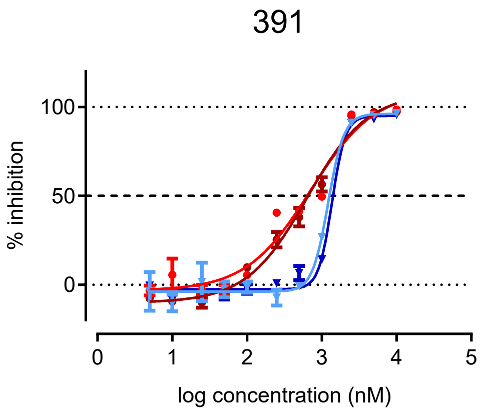

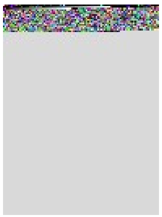

Figure 1. Percent inhibition of *P. falciparum* parasites (3D7 and Dd2 strains) by S-MGBs. IC_{50} curves calculated through a four-parameter logistic curve fitting in Prism (GraphPad).

4. *In Vitro* Cytotoxicity Assessment Against HEK293 For S-MGBs

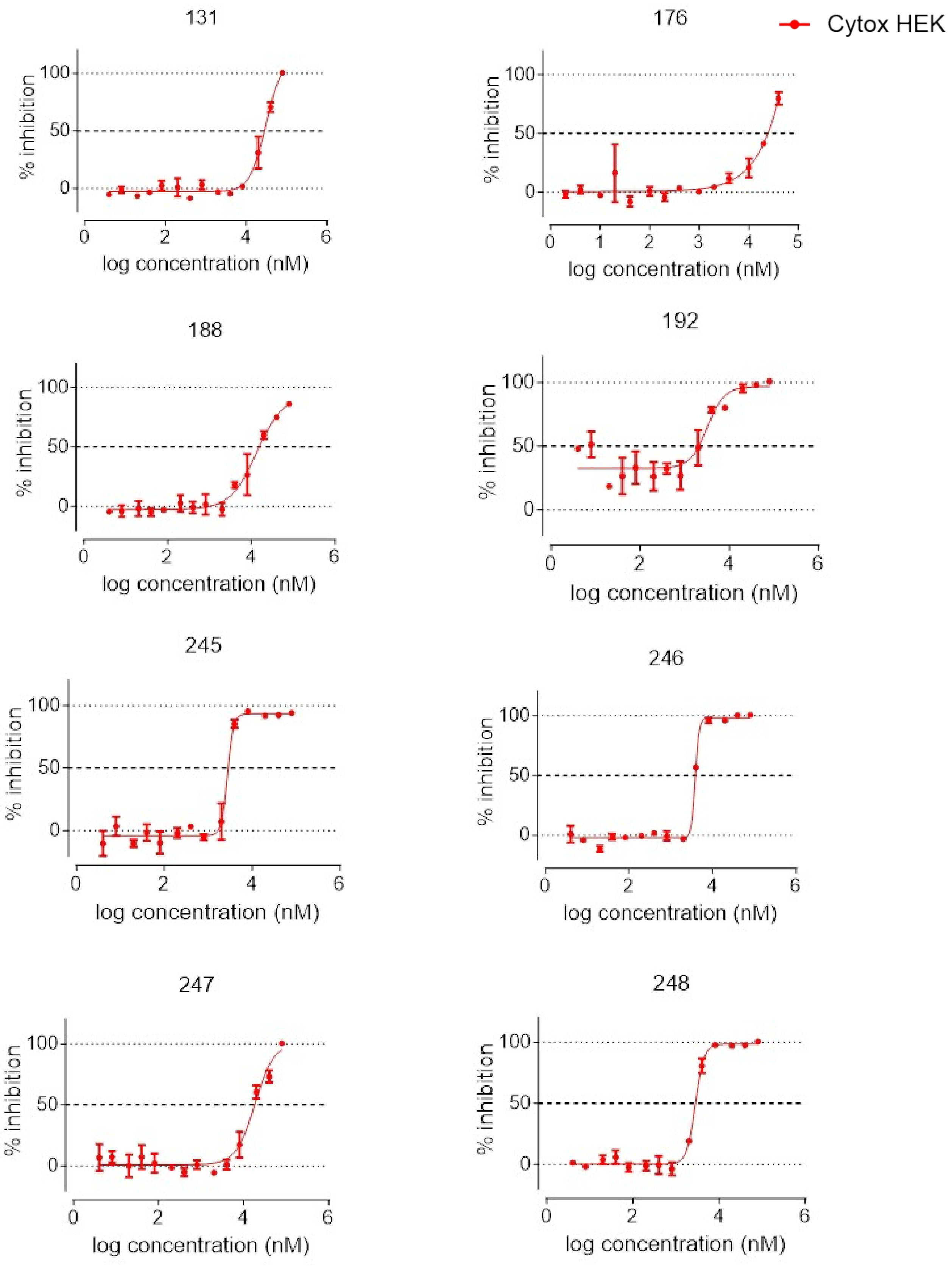

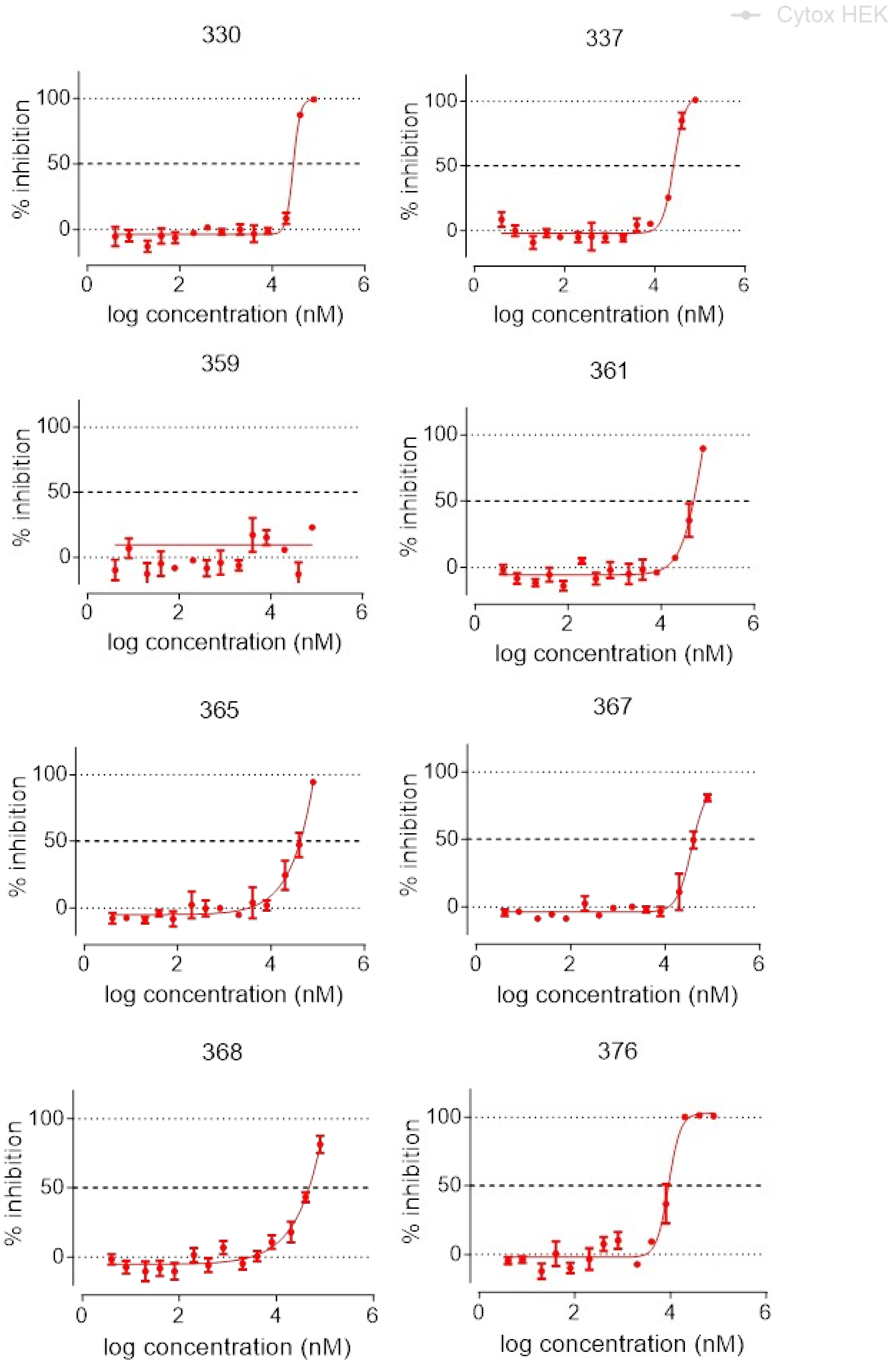
Cytox HEK
330
337
359
361
365
367
368
376
% inhibition
100
50
0
0
2
4
6
log concentration (nM)

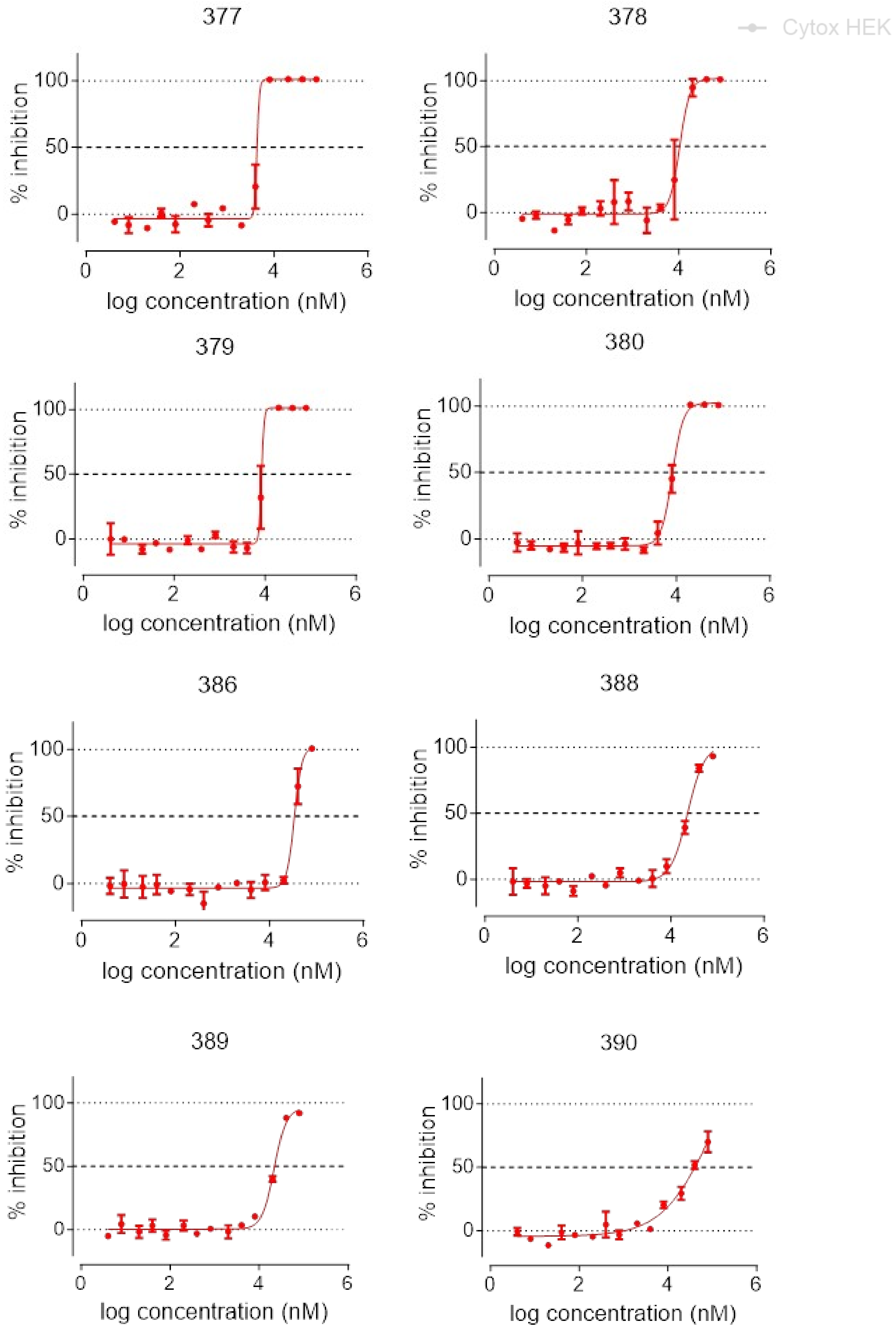

Cytox HEK
377
378
379
380
386
388
389
390
% inhibition
log concentration (nM)
100
50
0
0
2
4
6

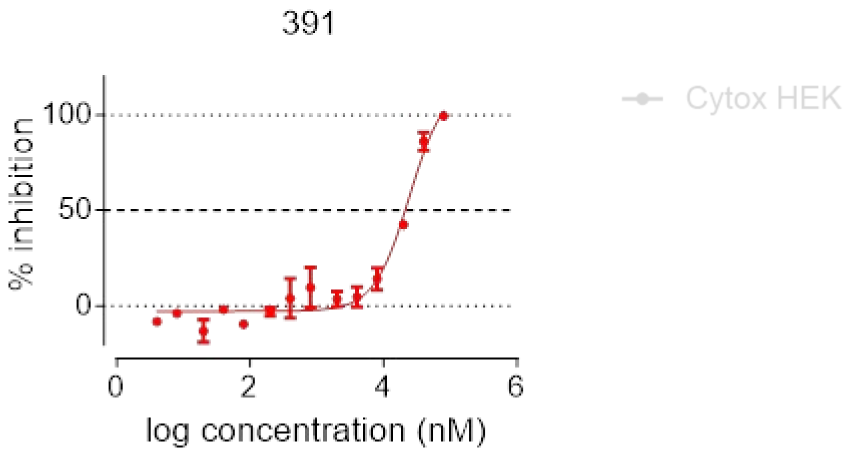

Figure 2. Percent inhibition of cell viability of Human Embryonic Kidney cells (HEK293) by S-MGBs. IC_{50} curves calculated through a four-parameter logistic curve fitting in Prism (GraphPad).

5. *In Vitro* Asexual *P. Falciparum* Assays Against 3D7 and Dd2, And Cytotoxicity Assessment Against HEK293 For Control Compounds

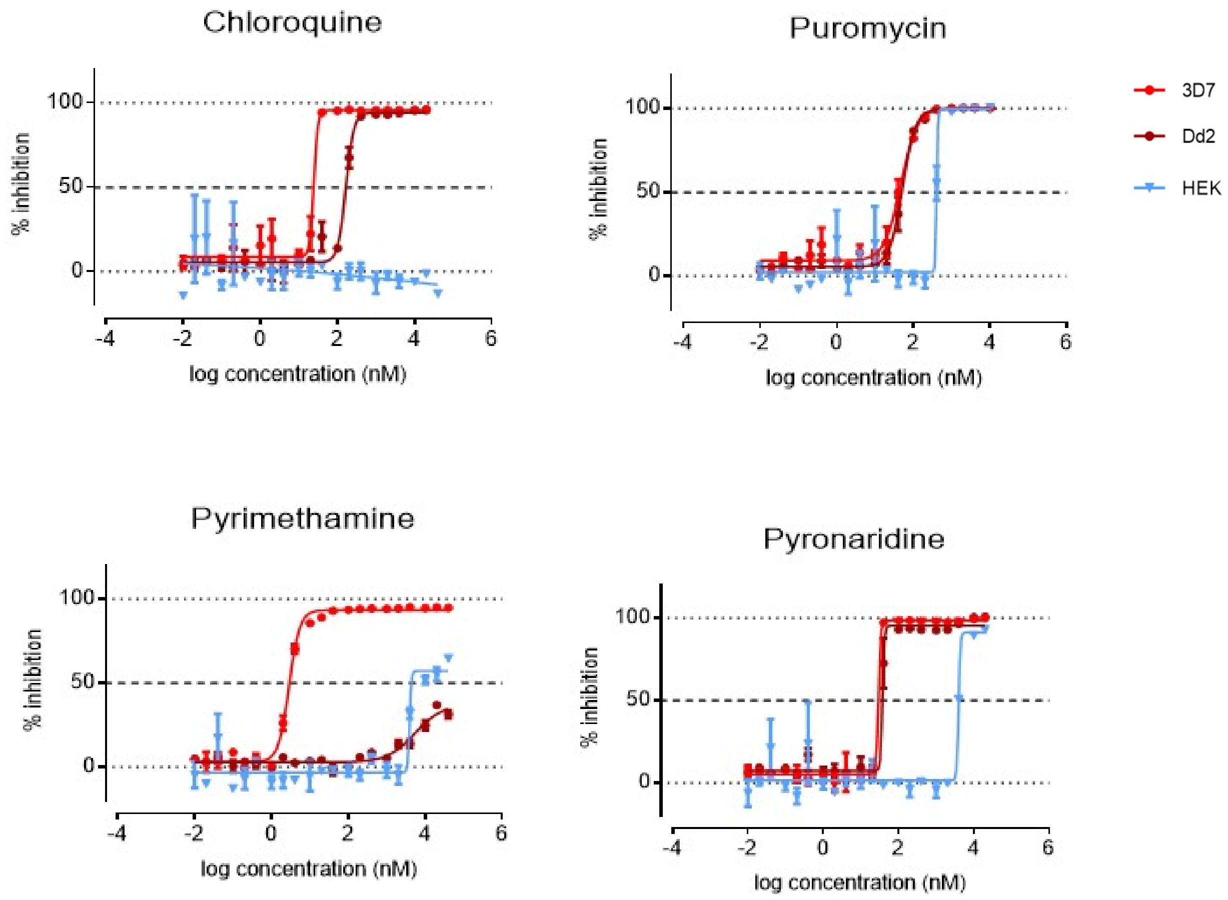

Figure 3. Percent inhibition of cell viability of Human Embryonic Kidney cells (HEK293) and Percent inhibition of *P. falciparum* parasites (3D7 and Dd2 strains) by control compounds. IC_{50} curves calculated through a four-parameter logistic curve fitting in Prism (GraphPad). by S-MGBs.

6. Native Mass Spectrometry

Species	m/z value	Calculated mass of neutral species (Da)
Single Stranded [SS]	3- : 1214.1<br>4- : 910.3	(1214.1*3) + 3 = 3645.3<br>(910.3*4) + 4 = 3645.2
Double Stranded [DS]	4- : 1821.7<br>5- : 1457.2	(1821.7*4) + 4 = 7290.8<br>(1457.2*5) + 5 = 7291.0
Double Stranded + 2 x S-MGB-365 [DS+2M]	4- : 2091.0<br>5- : 1672.6	(2106.0*4) + 4 = 8368.0<br>(1684.6*5) +5 = 8368.0

Table S2. Calculated and measured masses for each species observed in **Figure 4, Panel A** for DNA sequence 5'-CGCATATATGCG-3' **S-MGB-365.**

Species	m/z value	Calculated mass of neutral species (Da)
Single Stranded [SS]	3- : 1214.1<br>4- : 910.3	(1214.1*3) + 3 = 3645.3<br>(910.3*4) + 4 = 3645.2
Double Stranded [DS]	4- : 1821.7<br>5- : 1457.2	(1821.7*4) + 4 = 7290.8<br>(1457.2*5) + 5 = 7291.0
Double Stranded + 2 x S-MGB-368 [DS+2M]	4- : 2106.0<br>5- : 1684.6	(2106.0*4) + 4 = 8428.0<br>(1684.6*5) +5 = 8428.0

Table S3. Calculated and measured masses for each species observed in **Figure 4, Panel B** for DNA sequence 5'-CGCATATATGCG-3' **S-MGB-368.**

Species	m/z value	Calculated mass of neutral species (Da)
Single Stranded [SS]	3- : 1214.1<br>4- : 910.3	(1214.1*3) + 3 = 3645.3<br>(910.3*4) + 4 = 3645.2
Double Stranded [DS]	4- : 1821.7<br>5- : 1457.2	(1821.7*4) + 4 = 7290.8<br>(1457.2*5) + 5 = 7291.0
Double Stranded + 1 x S-MGB-359 [DS+1M]	5- : 1574.7	(1574.7*5) + 5 = 7877.0
*Double Stranded + 2 x S-MGB-359 [DS+2M]	4- : 2115.5<br>5- : 1692.2	(2115.5*4) + 4 = 8466.0<br>(1692.2*5) +5 = 8466.0

Table S4. Calculated and measured masses for each species observed in **Figure 4, Panel C** for DNA sequence 5'-CGCATATATGCG-3' **S-MGB-359.**
